# Erythropoietin Abrogates Post-Ischemic Activation of the NLRP3, NLRC4, and AIM2 Inflammasomes in Microglia/Macrophages in a TAK1-Dependent Manner

**DOI:** 10.1007/s12975-021-00948-8

**Published:** 2021-10-09

**Authors:** Ole Heinisch, Thomas Zeyen, Tobias Goldmann, Marco Prinz, Michael Huber, Jennifer Jung, Eren Arik, Shahin Habib, Alexander Slowik, Arno Reich, Jörg B. Schulz, Pardes Habib

**Affiliations:** 1 Department of Neurology, Medical Faculty, RWTH Aachen University, Pauwelsstraße 30, D-52074 Aachen, Germany; 2 Department of Neurology, University Hospital of Bonn, Bonn, Germany; 3 Institute of Neuropathology, Faculty of Medicine, University of Freiburg, Freiburg, Germany; 4 Signalling Research Centres BIOSS and CIBSS, University of Freiburg, Freiburg, Germany; 5 Center for Basics in NeuroModulation (NeuroModulBasics), Faculty of Medicine, University of Freiburg, Freiburg, Germany; 6 Institute of Biochemistry and Molecular Immunology, Medical Faculty, RWTH Aachen University, Aachen, Germany; 7 Medical Biochemistry, Department of Biochemistry, University of Leicester, Leicester, UK; 8 Department of Anatomy and Cell Biology, Medical Faculty, RWTH Aachen University, Aachen, Germany; 9 JARA-BRAIN Institute of Molecular Neuroscience and Neuroimaging, Forschungszentrum Jülich GmbH and RWTH Aachen University, Aachen, Germany

**Keywords:** Stroke, Microglia, Inflammasomes, TAK1, EPO, Neuroinflammation

## Abstract

Inflammasomes are known to contribute to brain damage after acute ischemic stroke (AIS). TAK1 is predominantly expressed in microglial cells and can regulate the NLRP3 inflammasome, but its impact on other inflammasomes including NLRC4 and AIM2 after AIS remains elusive. EPO has been shown to reduce NLRP3 protein levels in different disease models. Whether EPO-mediated neuroprotection after AIS is conveyed via an EPO/TAK1/inflammasome axis in microglia remains to be clarified. Subjecting mice deficient for TAK1 in microglia/macrophages (Mi/MΦ) to AIS revealed a significant reduction in infarct sizes and neurological impairments compared to the corresponding controls. Post-ischemic increased activation of TAK1, NLRP3, NLRC4, and AIM2 inflammasomes including their associated downstream cascades were markedly reduced upon deletion of Mi/MΦ TAK1. EPO administration improved clinical outcomes and dampened stroke-induced activation of TAK1 and inflammasome cascades, which was not evident after the deletion of Mi/MΦ TAK1. Pharmacological inhibition of NLRP3 in microglial BV-2 cells did not influence post-OGD IL-1β levels, but increased NLRC4 and AIM2 protein levels, suggesting compensatory activities among inflammasomes. Overall, we provide evidence that Mi/MΦ TAK1 regulates the expression and activation of the NLRP3, NLRC4, AIM2 inflammasomes. Furthermore, EPO mitigated stroke-induced activation of TAK1 and inflammasomes, indicating that EPO conveyed neuroprotection might be mediated via an EPO/TAK1/inflammasome axis.

**Graphical Abstract:**

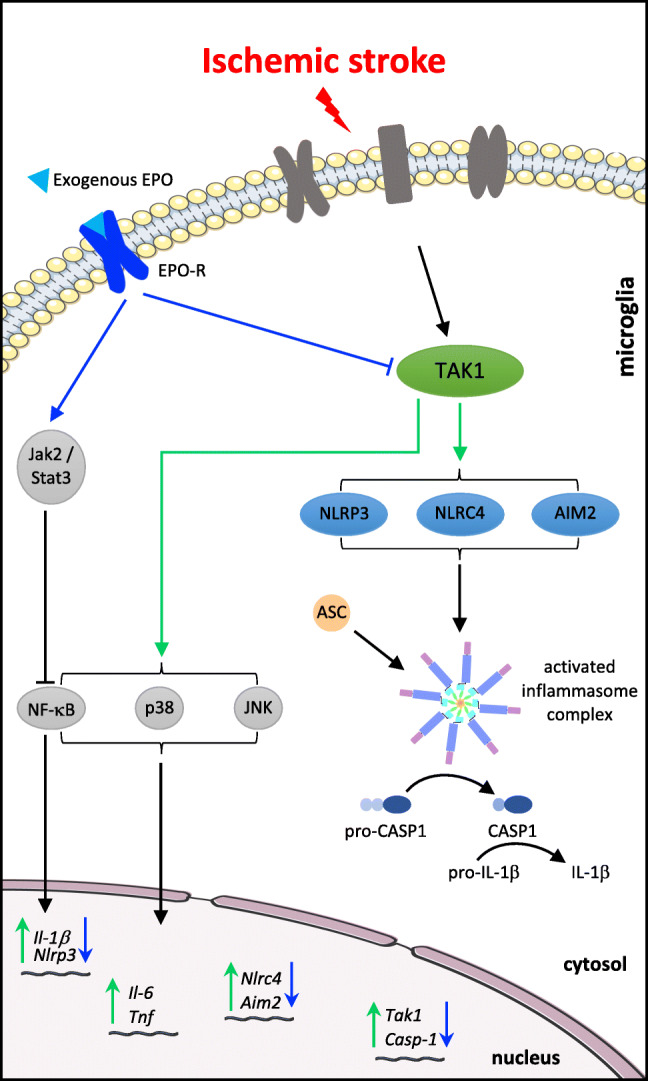

**Supplementary Information:**

The online version contains supplementary material available at 10.1007/s12975-021-00948-8.

## Introduction

Acute ischemic stroke (AIS) remains a leading cause of death and long-term disability worldwide and is associated with a high socioeconomic burden [1–3]. The current standard of care for AIS is thrombolysis by intravenous recombinant tissue plasminogen activator (rtPA, alteplase) within 4.5 h of stroke onset, and endovascular therapy in selected patients within 24 h of stroke onset [4–8]. However, due to their time-dependency and their strict eligibility criteria, both reperfusion modalities are only applicable to less than 15% of AIS patients [9, 10]. Currently, no neuroprotective/neurorestorative therapies have been approved highlighting the urgent need for more basic research.

Post-stroke neuroinflammation is one of the major contributors to secondary brain damage [11–13]. The inflammatory response is orchestrated by both brain residents and infiltrating immune cells. Microglial cells are the first responders to post-ischemic changes and account for 10 to 20% of all glial cells in the brain [14, 15]. Upon activation, depending on the duration and intensity of the stimulus, microglia either secrete pro-inflammatory cytokines resulting in further damage or promote tissue repair and synaptic remodeling through anti-inflammatory as well as neuro- and vasotrophic factors [13, 16–18]. Following AIS, activated (M1-phenotype) microglia produce pro-inflammatory mediators including tumor necrosis factor-α (TNF-α), interleukin (IL)-1β, and IL-6 [19]. The latter are regulated by the mitogen-activated protein kinase kinase kinase (MAP3K) TGFβ-activated kinase 1 (TAK1). TAK1 is highly abundant in the brain and predominantly expressed in microglial cells [20]. It controls viability and inflammation through multiple downstream effectors including MAP kinases p38 and JNK, as well as the transcription factor NF-κB [21–24]. Various stimuli including Toll-like receptor 4 (TLR4), TNF-α, IL-1β as well as hypoxia/ischemia have been shown to upregulate and activate TAK1 [20, 23, 25, 26]. While activation of TAK1 is reported to exacerbate brain damage, pharmacological inhibition of TAK1 using 5Z-7-Oxozeaenol exerts neuroprotection after subarachnoid hemorrhage as well as cerebral ischemia [23, 25, 26].

We have recently demonstrated that the deletion of microglial and macrophage (Mi/MΦ) TAK1 significantly reduced infarct sizes, neurological impairments, as well as the levels of TNF-α, IL-1β, and IL-6 in the periinfarct zone after transient middle cerebral artery occlusion (tMCAo) [20]. Although several cytokines are implicated in post-ischemic inflammation, a crucial role is particularly played by IL-1β. The maturation of IL-1β is executed by multiprotein complexes referred to as inflammasomes, which consist of cytoplasmic nucleotide-binding oligomerization domain (NOD)–like receptors (NLRs), the adaptor protein ASC (apoptosis speck–like protein containing a Caspase recruitment domain (CARD)) and pro-Caspase-1 [27, 28]. Upon activation, inflammasomes initiate the cleavage of pro-Caspase-1 to mature Caspase-1 (CASP1), which proteolytically activates the proinflammatory proteins IL-1β, IL-18, and Gasdermin-D leading to induction of inflammatory rapid cell death called pyroptosis [29, 30]. There is a large family of inflammasomes defined by different sensor proteins, and especially the NLRC4 (NLR family, CARD domain containing 4), AIM2 (absent in melanoma 2), and NLRP3 (NLR family, pyrin domain containing 3) inflammasomes have been shown to be associated with AIS [31–34]. NLRP3 is the most prominent microglial expressed inflammasome and is known to be regulated by TAK1 [35].

The hematopoietic growth factor erythropoietin (EPO) seems to have a regulatory impact on the aforementioned downstream effectors of TAK1. Although EPO has been approved for the treatment of renal anemia and has been successfully tested in multiple preclinical and clinical trials addressing different neurological diseases [36–42], the underlying mechanisms of EPO-conveyed cytoprotection remain poorly understood. EPO has been reported to abrogate the expression of NLRP3, IL-1β, and Caspase-1 in mouse models of asthma and lung injury [43, 44]. Here, EPO also mitigated the expression and activation of p38MAPK, TAK1, as well as the phosphorylation of NF-κB [43, 44]. This raises fundamental questions regarding mechanistic insights into the EPO-mediated cytoprotection: (i) Does TAK1, besides NLRP3, also regulate NLRC4 and AIM2 inflammasomes? (ii) Which role do NLRC4 and AIM2 play upon functional inhibition of microglial NLRP3 after cerebral ischemia? (iii) Is the EPO-mediated neuroprotection after stroke conveyed via an EPO/TAK1/inflammasomes axis?

To address these questions, we induced a tamoxifen-dependent conditional deletion of Mi/MΦ TAK1 utilizing *Cx3cr1*^*creER*^*-Tak1*^*fl/fl*^ mice and subjected these and the cre^ER^-negative Tak1^*fl/fl*^ control mice to 30 min of tMCAo followed by 6 and 72 h of reperfusion. EPO was administered directly, 24 and 48 h after tMCAo. Oxygen-glucose-deprivation (OGD) was performed in murine microglial BV-2 cells to assess the impact of TAK1- and NLRP3-inhibition on the NLRC4 and AIM2 inflammasomes. In total, our data indicate that EPO may convey neuroprotection via an EPO/TAK1/inflammasome axis.

## Materials and Methods

All experimental procedures were approved by the Animal Care Committee of the RWTH Aachen University and the District Government of North Rhine Westphalia in Recklinghausen, Germany (LANUV ID 84-20.04.2015.A292). All procedures were conducted in accordance with the ARRIVE guidelines. This study was not pre-registered.

### Animals

Mice were housed and handled according to the guidelines of the Federation for European Laboratory Animal Science Association (FELASA) in a pathogen-free, temperature-controlled (20–24 °C) facility with a 12/12-h light/dark cycle and access to pelleted food and water ad libitum. *Cx3cr1*^CreER^ mice (B6J.B6N(Cg)-Cx3cr1^tm1.1(cre)Jung^/J; Stock No: 025524, Jackson Laboratories) were generated on a C57BL/6 background [22]. Mice carrying loxP-site–flanked (floxed) alleles (Exon 2) of the TAK1-encoding gene *Map3k7* (*Tak1*^*fl/fl*^) were crossed with *Cx3cr1*^*CreER*^ mice (Jackson Laboratories). Littermates carrying the respective loxP-flanked alleles but the lacking expression of Cre recombinase (further referred to as “*Tak1*^*fl/fl*^”) served as control. To induce the Cre recombinase, 6 to 8-week-old mice were injected twice subcutaneously with 4 mg tamoxifen (TAM, Sigma-Aldrich, Taufkirchen, Germany) dissolved in 200 μL corn oil (Sigma-Aldrich, Taufkirchen, Germany) in a period of 48 h. The mice were bred by the Institute for Laboratory Animal Science and Experimental Surgery, Faculty of Medicine, RWTH Aachen University.

### Animal Surgery—Transient Middle Cerebral Artery Occlusion

Transient middle cerebral artery occlusion (tMCAo) or sham surgery was performed for 30 min followed by 6 or 72 h of reperfusion as previously described [45]. In brief, anesthesia was induced by 3% isoflurane in nitrous oxygen (30% O_2_ / 67% N_2_O) and was maintained with 1% isoflurane. The regional cerebral blood flow (rCBF) was monitored with a laser Doppler probe (Moor Instruments VMS-LDF2, Axminster, UK) above the left MCA territory (online resource 1C). In a supine position, the left common carotid artery (CCA) and the external carotid artery (ECA) were isolated and ligated. A silicon-coated filament (#602212PK10Re, Doccol, Sharon, MA, USA) was threaded into the internal carotid artery (ICA) for MCA occlusion. The filament was not inserted in the sham-treated animals. A reduction to <20% of the baseline in the rCBF was regarded as a sufficient occlusion of the MCA. During the procedure, the body temperature was maintained at 37 °C ± 0.5 °C using a feedback-controlled heating pad. After 30 min of tMCAo, the mice were supplemented with 0.5 mL saline i.p. Monitoring the regional cerebral blood flow to assure a sufficient MCA occlusion during every surgery revealed no significant differences between both genotypes (online resource 1C). The animals were then placed into temperature-controlled cages for the post-surgical survival period. The body weight and temperature were measured daily. For pain relief, buprenorphine (0.5 mg/kg body weight) was injected s.c. directly after surgery and every 8 h over the whole observation period. The primary endpoint of this study was the infarct size after 6 and 72 h of reperfusion. Decapitation of mice was performed after isoflurane overdose. All surgical procedures were performed from 8:00 to 11:30 a.m.

### Study Protocol

In this preclinical randomized and blinded controlled trial we subjected a total of 64 male mice (32 *TAK1*^*fl/fl*^, 32 *Cx3cr1*^*creER*^*-Tak1*^*fl/fl*^) to either 30 min of tMCAo or sham surgery followed by a reperfusion period of 6 or 72 h (online resource 1A, B). The study design is shown in the timeline diagram in online resource 1A. We have previously demonstrated that tamoxifen does not affect infarct sizes or neurological outcomes compared to the corresponding corn oil control [20]. Therefore, in this study, both genotypes were injected with tamoxifen at 6–8 weeks of age. At the age of 10–12 weeks, all mice were subjected to 30 min of tMCAo or sham surgery followed by a reperfusion period of 6 or 72 h. Clinical outcomes were monitored at six different time points (−1, 1, 6, 24, 48, and 72 h post-surgery) during the reperfusion phase. We included only male mice to exclude the neuroprotective impact of gonadal steroid hormones as previously described [46, 47]. The primary endpoint of this study was the evaluation of infarct sizes 6 and 72 h after surgery.

### Exclusion Criteria

We excluded mice from the study if a reduction in the rCBF was not lower than 80% of the baseline value and if recovery of the rCBF at CCAo level (60–70% of baseline) after 5 to 10 min reperfusion was absent. Furthermore, animals with brain hemorrhage, seizures, extensive weight loss (>20% of baseline), missing infarction in TTC-staining (described below) and those that did not develop sufficient neurological deficits (mNSS <5) were excluded. Mice that died during the post-surgical observation period (72 h) were excluded from all analyses, except the mortality rate between genotypes and treatment.

### EPO Administration

Recombinant human EPO (rhEPO) (Epoetin alfa Hexal, Hexal, Holzkirchen, Germany) was diluted in 0.9% NaCl. EPO in a dosage of 5000 U/kg body weight or 0.9% NaCl (vehicle control) was injected intra-peritoneally directly after tMCAo, 24 and 48 h after reperfusion as previously described [45]. In the analysis of the complete blood count, the three-times administration of EPO did not yield a significant difference in hematocrit (HCT), hemoglobin (HGB), and red blood cell count (RBC) compared to the vehicle (NaCl) group (Table 1).

**Table 1:** Demographics and hematology after 30 min of tMCAo followed by a reperfusion period of 72 h

Genotype	*Tak1*^*fl/fl*^				*Cx3cr1*^*creER*^*-Tak1*^*fl/fl*^			
Demographics								
Treatment [0.9% NaCl or 5000 U EPO/kg bodyweight]	NaCl		EPO		NaCl		EPO	
Reperfusion time	6 h	72 h	6 h	72 h	6 h	72 h	6 h	72 h
Age [weeks]	11 (±1)	11 (±1)	11 (±1)	11 (±1)	11 (±1)	11 (±1)	11 (±1)	11 (±1)
Sex [female/male]	Male	Male	Male	Male	Male	Male	Male	Male
Bodyweight [g]	27.47 (±0.97)	26.08 (±1.73)	26.28 (±1.39)	27.15 (±2.10)	25.80 (±1.60)	26.47 (±1.95)	26.52 (±1.48)	26.32 (±1.65)
Hematology								
Genotype	*Tak1*^*fl/fl*^				*Cx3cr1*^*creER*^*-Tak1*^*fl/fl*^			
Treatment [0,9% NaCl or 5000 U EPO/kg bodyweight]	NaCl		EPO		NaCl		EPO	
Reperfusion time	6 h	72 h	6 h	72 h	6 h	72 h	6 h	72 h
WBC [10^3^/μL]	3.43 (±1.68)	4.40 (±1.72)	3.85 (±1.79)	4.37 (±2.72)	0.05 (±1.45)	5.92 (±1.98)	3.86 (±1.26)	5.60 (±1.63)
RBC [10^6^/μL]	9.93 (±0.98)	9.56 (±1.43)	9.26 (±1.08)	9.17 (±1.52)	9.77 (±1.53)	9.52 (±0.83)	9.38 (±1.24)	9.78 (±1.02)
HGB [g/dL]	14.77 (±1.59)	14.30 (±2.04)	14.37 (±1.68)	13.53 (±2.04)	14.65 (±0.75)	14.07 (±1.08)	13.74 (±1.34)	14.58 (±1.44)
HCT [%]	42.23 (±4.52)	40.07 (±5.86)	40.38 (±4.18)	38.50 (±6.13)	40.70 (±3.70)	39.72 (±2.91)	42.05 (±5.16)	41.38 (±4.18)
MVC [fl]	42.47 (±0.31)	42.02 (±1.21)	42.47 (±1.43)	42.05 (±0.25)	42.80 (±0.50)	41.75 (±0.68)	42.85 (±0.94)	42.35 (±1.28)
MCH [pg]	14.87 (±0.21)	15.00 (±0.43)	14.96 (±0.44)	14.80 (±0.29)	15.10 (±0.34)	14.80 (±0.22)	15.68 (±0.39)	14.92 (±0.45)
MCHC [g/dL]	34.97 (±0.34)	35.70 (±0.36)	35.53 (±0.29)	35.18 (±0.54)	35.15 (±0.35)	35.40 (±0.33)	35.36 (±0.76)	35.27 (±0.46)
PLT [10^3^/μL]	706.33 (±194.44)	783.83 (±268.01)	826.58 (±238.74)	1101.17 (±540.81)	435.50 (±260.50)	866.00 (±196.61)	794.64 (±345.96)	968.83 (±343.00)

Data are presented in mean ± SD (*n* = 6). Abbreviations: *WBC* white blood cells; *RBC* red blood cells; *HGB* hemoglobin; *HCT* hematocrit; *MVC* middle corpuscular volume; *MCH* middle corpuscular hemoglobin; *MCHC* middle corpuscular hemoglobin concentration; *PLT* platelets

### Assessment of Neurological Outcomes—Modified Neurologic Severity Score

To evaluate the general status and focal neurological impairments after 30 min of tMCAo or sham surgery, a modified neurologic severity score (mNSS) was utilized [37, 48]. This test battery separately grades motor function (body asymmetry, muscle status, abnormal movement, and gait), sensory function (visual, tactile, and proprioceptive), and reflexes (corneal reflex, pinna reflex, whisker response to light touch, startle reflex). The score ranges from 0 (no deficits) to 15 points representing the poorest performance in all items and is calculated as the sum of the general and focal deficits. For the inability to perform the task, abnormal performance, or lack of a tested reflex, one point was awarded. We defined a score of 1 to 5 as mild, 6 to 10 as moderate, and 11 to 15 as severe neurological deficits. Two individual investigators, other than the experimenter and blinded to genotype and treatment of the mice, examined each animal 1 h before and 1, 6, 24, 48, and 72 h after tMCAo or sham surgery.

### Evaluation of Infarct Sizes and Hematology

Infarct volumes were measured using the 2,3,5-triphenyltetrazolium chloride (TTC) staining method as previously described [20, 37, 45]. After a reperfusion period of 6 or 72 h, mice were deeply anesthetized and EDTA blood of each animal was taken transcardially for a blood count using the Celltac α MEK-6450 K (Nihon Kohden Europe, Rosbach, Germany) in the Institute for Laboratory Animal Science and Experimental Surgery, Faculty of Medicine, RWTH Aachen University, RWTH Aachen University (Table 1). The brains were removed immediately and sliced coronally. The 1-mm-thick brain sections were incubated in 2% TTC (Sigma-Aldrich, Taufkirchen, Germany) for 10 min at 37 °C followed by a fixation in 10% formaldehyde in phosphate-buffered saline (PBS). The stained sections were photographed (Fujifilm X-T20, XF18-55 mm) and evaluated in a blinded manner using ImageJ software (NIH, Bethesda, Md., USA). The infarct volumes were corrected for brain edema by using Reglodi’s method: Adjusted-lesion size = measured lesion × (contralateral hemisphere/ipsilateral hemisphere). Total infarct volumes were calculated by adding the mean area of each section and multiplied by the thickness of the sections.

### BV-2 Cell Line, Cultivation, and Oxygen Glucose Deprivation

The murine microglial BV-2 cell line was originally generated by Blasi and colleagues [49] and has been used in oxygen glucose deprivation (OGD) experiments in our previous studies [47, 50]. BV-2 cells were maintained in a humidified environment at 37 °C with 5% CO_2_. The cells were cultured in Dulbecco’s modified Eagle medium (DMEM, Pan Biotech GmbH, Aidenbach, Germany) supplemented with 10% fetal bovine serum (FBS, Pan Biotech GmbH, Aidenbach, Germany) and 0.5% penicillin–streptomycin (PS, Pan Biotech GmbH, Aidenbach, Germany). Cells were sub-cultured at a level of approx. 80% confluence. Trypsin/EDTA (Pan Biotech GmbH, Aidenbach, Germany) treatment was used to detach cells for splitting.

To mimic ischemic stroke in vitro, we performed an oxygen glucose deprivation (OGD) as previously described [20, 37]. After determining the optimal doses (online resource 3C,D), the inhibitors 5Z-7-Oxozeaenol [100 nM] (5Z-7-Oxo); (Merck-Millipore Darmstadt, Germany) and MCC950 [1 μM]; (AdipoGen Life Sciences, Liestal, Switzerland) were added to the cells 1 h prior to OGD in a customized hypoxia chamber (Fig. 7A) (Part#,: C174, C21, Biospherix, Parish, NY, USA). ODG was performed for a total duration of 90 min including 30 min of severe hypoxia (<1% O_2_). We monitored the oxygen levels (#200001735, PreSens, Regensburg, Germany), temperature (set at 37 °C), as well as the pressure (online resource 3B) in the chamber and in the cell medium during the entire ODG periods. Normoxic controls were maintained at 37 °C, 5% CO_2_, and atmospheric pressure.

### Cell Viability

Viability of the cells was assessed by cell counting (Roche Innovatis Cedex XS, Basel, Schweiz). In addition, secreted lactate dehydrogenase (LDH) levels were measured in the cell supernatant using the CytoTox 96 Non-Radioactive Cytotoxicity Assay (#G1782, Promega, Madison, WI, USA) according to the manufacturer’s protocol. A lysis-control after application of Lysis solution (Triton X-100) was used as a positive internal control.

### Reverse-Transcription Quantitative PCR

Gene expression analyses were performed with brain tissue biopsies from the peri-infarct areas (Bregma 0 ± 1 mm) using a stereomicroscopic approach. After dissolving and homogenizing the tissue in PeqGold (#30–2010, PeqLab, Germany), the total RNA was extracted using peqGold RNA TriFast as previously described [20, 37]. Complementary DNA was synthesized with the MMLV reverse transcription kit (Cat.# 28025-013, Thermo Fischer Scientific, Waltham, MA, USA) and random hexanucleotide primers (Cat.# 48190-01, Thermo Fischer Scientific, Waltham, MA, USA) using 1 μg of total RNA. Triplicates of every sample were transferred by a pipetting robot (Corbett CAS-1200, Qiagen, Hilden, Germany) to Rotor-Gene strip reaction tubes (I1402–0400, Starlab, Hamburg, Germany) and RT-qPCR analysis was performed using the Rotor-Gene Q device (Qiagen, Hilden, Germany). RNase-free H_2_O (Merck-Millipore Darmstadt, Germany) served as no template control (NTC) and primer efficiencies were calculated using the Pfaffl method [51]. The target genes and two housekeeping genes, hypoxanthine guanine phosphoribosyltransferase (*Hprt)* and glyceraldehyde-3-phosphate dehydrogenase (*Gapdh*) were measured at cycle threshold (Ct values) and relative quantification was calculated by the ΔΔCt method using the qbase+ software (Biogazelle, Belgium). A list of used primers is given in Table 2.

**Table 2:** List of Primers used for RT-qPCR

Target gene	Forward (fwd)	Reverse (Rev)	AT [°C]	Species
Aim2	GATTCA AAGTGCAGGTGCGG	TCTGAGGCTTAGCTTGAGGAC	61	Mouse
Asc	AGTCTGGAGCTGTGGCTACTGC	TGAGTGCTTGCCTGTGTTGGTC	60	Mouse
Casp1	CGCATTTCCTGGACCGAGTGG	GAGGGCAAGACGTGTACGAGTG	59	Mouse
Gapdh	ATGTTCCAGTATGACTCCACTCACG	GAAGACACCAGTAGACTCCACGACA	60	Mouse
Hprt	GCTGGTGAAAAGGACCTCT	CACAGGACTAGAACACCTGC	62	Mouse
II-18	GCCTGTGTTCGAGGATATGACT	CCTCACAGAGAGGGTCACAG	60	Mouse
II-1β	GCACTACAGGCTCCGAGATGAAC	TTGTCGTTGCTTGGTTCTCCTTGT	61	Mouse
Map3k7	CGGAAGAGGAGCTTTTGGAGT	GGTTCACACGTGACAACTGC	59	Mouse
Nlrc4	CAGGTGGTCTGATTGACAGC	CCCCAATGTCAGACAAATGA	60	Mouse
Nlrp3	GATCCTGACAACACGCGGA	CCTGGGGGACTTTGGAATCAG	61	Mouse

Sequences, annealing temperature (AT) and species are given

### Western Blotting

Protein analysis was performed by western blot as previously described [20]. In brief, tissue samples were lysed in ice-cold Radioimmunoprecipitation assay (RIPA) buffer containing a proteinase and phosphatase inhibitor cocktail (#11873580001, Roche, Basel, Schweiz). Protein concentration was measured using a BCA-kit (Biorad, Feldkirchen, Germany). SDS-PAGE was performed under reducing conditions, followed by blotting transfer on a polyvinylidene difluoride (PVDF) membrane. Blocking of unspecific binding was performed with 5% skim milk (#T145.3, Roth, Karlsruhe, Germany) or 5% bovine serum albumin (#8076.4, Roth, Karlsruhe, Germany) in Tris-buffered saline with 0.05% Tween20 (#9127.1, Roth, Karlsruhe, Germany) (TBS-T) for 30 min at room temperature (RT). The primary antibody was incubated in TBS-T or 5% BSA/TBS-T at 4 °C overnight, followed by the incubation of the appropriate secondary antibody in TBS-T at RT for 1 h. Visualization of the immunoreactivity was performed by enhanced chemiluminescence (Thermo Fischer Scientific, Waltham, Massachusetts, USA). Actin served as a loading control. Densitometric measuring was performed using ImageJ software (NIH, Bethesda, MD, USA). A list of antibodies and their dilution is given in Table 3.

**Table 3:** List of antibodies used for western blot

Antibody	MW [kDA]	Host	Manufacturer	Catalog number	Dilution	Method
ACTIN	43	Mouse	Abcam	ab3280	1:1000	WB
AIM2	39	Rabbit	Bioss Antibodies	BS-5986R	1:1000	WB/IF
ASC	24	Mouse	Santa Cruz Biotechnology	sc-514414	1:1000	WB
CASP1	48	Rabbit	Thermo Scientific	PA 587536	1:1000	WB
HIF1a	93	Rabbit	Novusbio	NB100-479	1:1000	WB
IL-1β	17	Rabbit	Novusbio	NBP1-42767	1:1000	WB/IF
NLRC4	116	Rabbit	Thermo Scientific	PA5-72978	1:1000	WB/IF
NLRP3	118	Rabbit	Thermo Scientific	PA5-79740	1:1000	WB
pTAK1	82	Rabbit	Cell signaling	9339	1:1000	WB
TAK1	70	Mouse	Santa Cruz Biotechnology	sc-7968 sample	1:1000	WB
NeuN		Mouse	Merck-Millipore	MAB377	1:1000	IF
GFAP		Mouse	Merck-Millipore	MAB3402	1:500	IF
IBA-1		Goat	Novusbio	NB100-1028	1:500	IF
Anti-mouse IgG			GE Healthcare	NXA931V	1:10000	WB
Anti-rabbit IgG			GE Healthcare	NA934	1:10000	WB
Goat anti-rabbit			Invitrogen	A11008	1:500	IF
Goat anti-mouse			Invitrogen	A11032	1:500	IF
Donkey anti-rabbit			Invitrogen	A21206	1:500	IF
Donkey anti-goat			Invitrogen	A11058	1:500	IF

Molecular weight (MW), host, manufacturer, catalog number, dilution, and the method used are given

### Immunohistochemistry

After euthanasia, the brains were fixed with 4% paraformaldehyde (PFA) before the paraffin-embedding process. Staining was performed on 5-μm sections from Bregma ±0 regions. First, the sections were deparaffinated and incubated for 10 min with citrate buffer (pH 6.0) in a microwave. The slices were blocked for 30 min followed by incubation with the first primary antibody at 4 °C overnight. Then the first secondary antibody was incubated for 1 h at RT. Afterward, the second primary antibody was incubated for 3 h at RT followed by the second secondary antibody for 1 h at RT and DAPI (Roth, Karlsruhe, Germany). We counted double-positive cells in twelve selected fields and expressed the percentage of double-positive cells/100 cells in the (peri-) infarct zone. A list of the used antibodies is given in Table 3.

### Immunocytochemistry

After removal of the medium, cells were fixed with 3.7% Formaldehyde in PBS for 30 min at RT and washed three times with PBS. Then, cells were permeabilized by incubation with 0.2% Triton X-100 in PBS for 10 min at RT, followed by blocking with IFF buffer for 1 h. The primary antibody diluted in PBS was applied and incubated overnight at 4 °C. The following day the secondary antibody diluted in PBS was applied for 1 h at RT. In addition, cell nuclei were stained with 4′,6-Diamidin-2-phenylindol (DAPI) (Roth, Karlsruhe, Germany). Immunocytochemical staining was performed using antibodies against Ionized calcium-binding adaptor molecule 1 (IBA1) to detect microglia. Visualization was performed with a Leica fluorescence microscope (Leica, Wetzlar, Germany). A list of antibodies used in this study is given in Table 3.

### Caspase-1 Activity Assay

Caspase-1 (CASP1) activity was determined in the cells and the supernatant using the Caspase-Glo® 1 Inflammasome Assay (#G9951, Promega, Madison, WI, USA) according to the manufacturer’s instructions. The luminescence was measured using the multiplate reader (Tecan GmbH, Switzerland).

### Enzyme-Linked Immunosorbent Assay

IL-1β enzyme-linked immunosorbent assay (ELISA) was performed according to manufacturers’ protocols and as described previously [20] (IL-1β Mouse Uncoated ELISA Kit Invitrogen, #88-7013-88, Thermo Fischer Scientific, Waltham, MA, USA). In brief, the plates were coated with the coating antibody and incubated overnight at 4 °C. Then, blocking was performed for 2 h at RT, followed by the loading of the wells with 100 μL of each sample and internal standard control which were then incubated overnight at 4 °C. Next, the detection antibody was added for 2 h at RT, followed by enzyme (Avidin-HRP) incubation for 30 min at RT. Lastly, the substrate (TMB Solution) was added, and the plate was measured at 450 nm in the Tecan reader (Tecan GmbH, Switzerland).

### Statistics

The investigators/surgeons were blinded to genotype and experimental groups during experiments and analysis. Animals were identified by a technical assistant not involved in the analyses by earmarks that assigned numbers to each, which were announced to the investigator only after finishing experiments and analysis. Randomization was carried out using sorting by random numbers (QuickCalcs, GraphPad prism 6.0). Data analysis and visualization were performed using GraphPad Prism (version 8.4.3, San Diego, CA, USA). Residuals were analyzed for normal distribution using the Shapiro-Wilk and D’Agostino-Pearson omnibus normality test. Variance homogeneity was tested using the Bartlett test or the Spearman’s rank correlation test for heteroscedasticity. For the identification of outliers, a ROUT test was utilized. In case of significances in normality and/or variance homogeneity, values were BOX-COX-transformed after calculation of the optimal lambda and used for statistical analysis. Intergroup differences were tested by ANOVA two-way or three-way followed by Tukey post hoc test (multiple groups). Data are given as arithmetic means ± SD. *p* < 0.05 was considered statistically significant. Asterisks indicate significance between-group differences, “#” compares EPO vs. vehicle, and “§” compares differences of each genotype in the course of time. We have previously demonstrated that in TAK1^fl/fl^ mice (108.52 mm^3^ ± 4.03 mm^3^) infarct sizes were twice as large compared to Cx3cr1^creER^-Tak1^fl/fl^ mice (58.4 mm^3^ ± 2.93 mm^3^) [20]. Here, we consider 20% differences in infarct sizes as significant. Therefore, we required a minimum of 4 animals per group to detect such a difference at 95% confidence (*a* = 0.05), expected attrition of less than 5%, and a power of 80%. Power analysis was carried out with G*Power. The number of animals, technical, and experimental repeats are indicated in the corresponding figure legends including the statistical tests employed.

## Results

### Both EPO and Deletion of Mi/MΦ TAK1 Reduced Infarct Sizes and Neurological Impairments after Ischemic Stroke

**Fig. 1:**
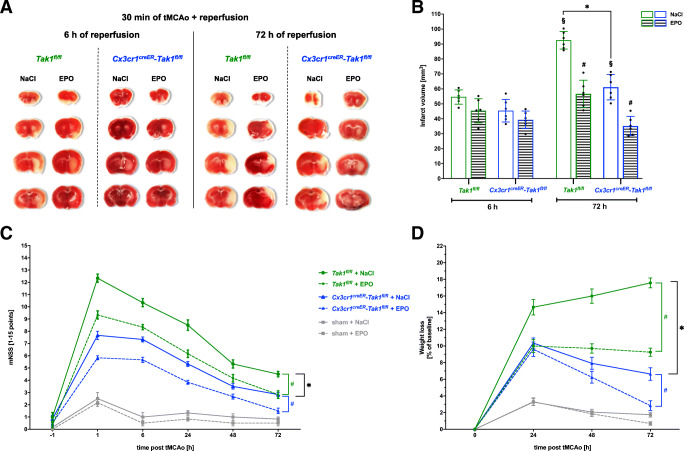
Mi/MΦ TAK1 deletion and administration of EPO reduced infarct sizes and neurological impairment after stroke**. A** Representative images of TTC-stained brain slices of both genotypes after tMCAo. Vital tissue is stained red, while apoptotic/necrotic tissue remains white. **B** Post-ischemic computer-assisted infarct volumetry of *Tak1*^*fl/fl*^ and *Cx3cr1*^*creER*^*-Tak1*^*fl/fl*^ mice after 6 and 72 h of reperfusion. **C** Modified neurologic severity score (mNSS) in a time-dependent manner (−1 to 72 h) was assessed to grade neurological outcome after stroke or sham surgery. **D** Weight loss after tMCAo or sham surgery in the observation period of 72 h. Each experiment was conducted with *n* = 4 sham mice, 6 *Tak*^*fl/fl*^*, and 6 Cx3cr1*^*creER*^*-Tak1*^*fl/fl*^ mice per treatment group and time-point. For statistical analysis, a 3-way ANOVA followed by Tukey’s multiple comparisons test was performed. Bars represent means ± SD. *p < 0.05 intergroup or treatment comparison, § comparison of each group at different time-points #*p* < 0.05 EPO treatment compared to the NaCl group

In the present study, we used TAK1-deficient (*Cx3cr1*^CreER^ *Tak*^*fl/fl*^) mice and respective control mice (*Tak*^*fl/fl*^) to study the effect of TAK1 and Erythropoietin (EPO) on inflammasome contribution to AIS. The primary endpoint of the study was the evaluation of infarct sizes in TTC-stained brain sections of both genotypes after 6 or 72 h of reperfusion (Fig. 1A). After 6 h of reperfusion, we observed predominantly striatal infarctions in both genotypes, with no difference in their extent between genotypes. After 72 h of reperfusion, the infarct sizes in *Tak1*^*fl/fl*^ mice almost doubled with a clear expansion and allocation in the cortical area (mean ± SD = 92.5 ± 5.8 mm^3^, *p* < 0.001) (Fig. 1B). In contrast, the Mi/MΦ TAK1-deficient mice exhibited an increase in infarct size of approximately 35% after 72 h compared to the early reperfusion phase (mean ± SD = 61.2 ± 8.5 mm^3^, *p* = 0.0095). We also detected a comparable reduction in infarct sizes after EPO administration. Although EPO application evoked a tendency of reduction of infarct sizes in both genotypes after 6 h of reperfusion, an almost 50% reduction of infarct sizes was observed after 72 h of reperfusion in the control animals (mean SD = 56.7 ± 9.0 mm^3^, *p* < 0.001). EPO in *Cx3cr1*^*creER*^*-Tak1*^*fl/fl*^ mice led to a further reduction of infarct volumes by 63% (mean SD = 35.2 ± 6.4 mm^3^, *p* < 0.001), suggesting a synergistic effect of EPO and deletion of Mi/MΦ TAK1 (Fig. 1B).

The comparably larger infarct sizes in the *Tak1*^*fl/fl*^ mice were also associated with higher post-ischemic neurological impairments and weight loss. While the sham group (mild injury, 0–4 points) recovered rapidly from surgery and exhibited hardly any deficits during the entire observation period, tMCAo in both genotypes caused considerable neurological deficits, especially in the initial phase of the reperfusion (up to 6 h post-stroke) (Fig. 1C). The deletion of Mi/MΦ TAK1 (moderate injury 5–9 points) showed significantly lower deficits over the entire 72 h of reperfusion (*p* < 0.01) compared to the control animals (severe injury 10–15 points). Both *Tak1*^*fl/fl*^ (*p* < 0.05) and *Cx3cr1*^*creER*^*-Tak1*^*fl/fl*^ mice (*p* < 0.01) gained clinical benefits from EPO administration over the entire observation period of 72 h after stroke. The EPO-dependent beneficial clinical outcomes were also reflected in the weight measurement since both genotypes displayed significantly lower weight loss compared to the vehicle group (*p* < 0.001) (Fig. 1D).

These data suggest that both EPO and the deletion of Mi/MΦ TAK1 reduce infarct sizes and improve clinical outcomes. However, EPO-mediated protective effects do not appear to depend fully on Mi/MΦ TAK1, but the combination of EPO and Mi/MΦ TAK1 deletion seems to synergistically provide greater cytoprotection.

### Post-Ischemic Upregulated mRNA Levels of the Inflammasomes *Nlrp3*, *Aim2*, and *Nlrc4* as well as Their Downstream Cascade Were Attenuated by EPO

**Fig. 2:**
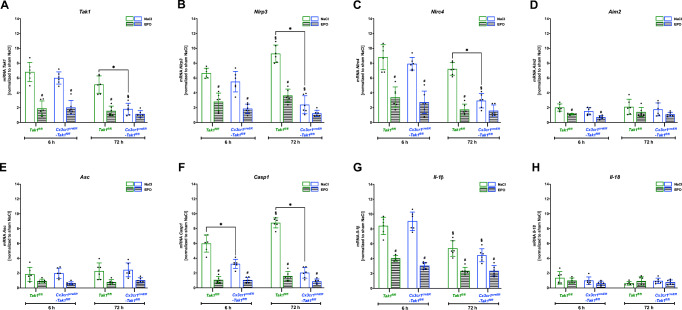
Post-stroke elevated mRNA levels of *Tak1* and the inflammasomes *Nlrp3*, *Aim2*, *Nlrc4* as well as their downstreaming cascade were abrogated by EPO. Brain biopsies taken from the peri-infarct-zone and the corresponding contralateral hemispheres of both genotypes after 6 and 72 h of reperfusion were utilized for gene expression analysis using RT-qPCR. **A–H** Post-ischemic mRNA levels of *Tak1, Nlrp3,* Nlrc4, *Aim2, Pycard (Asc)*, *Casp1, Il-1β*, and *Il-18* (*n* = 6). A 3-way ANOVA followed by Tukey’s multiple comparisons test was performed. Bars represent means ± SD. *p < 0.05 intergroup or treatment comparison, § comparison of each group at different time-points, #*p* < 0.05 EPO treatment compared to the NaCl group

To investigate the impact of EPO and the depletion of Mi/MΦ TAK1 on gene expression of the inflammasomes as well as their associated downstream cascade after an ischemic stroke, we utilized brain biopsies from the periinfarct-zone of both genotypes after 6 and 72 h of reperfusion. Here we found a significant increase in the mRNA levels of *Tak1* and the inflammasomes *Nlrp3*, *Nlrc4*, and *Aim2* after stroke compared to sham surgery (Fig. 2A–D). Comparing *Tak1*^*fl/fl*^ with *Cx3cr1*^*creER*^*-Tak1*^*fl/fl*^ mice, both genotypes exhibited a significant upregulation of *Tak1* mRNA levels after 6 h of reperfusion, which was considerably reduced by EPO administration (*p* = 0.039) (Fig. 2A). However, while *Tak1* mRNA in the *Tak1*^*fl/fl*^ mice remained elevated after 72 h after tMCAo, Mi/MΦ TAK1 depletion resulted in a significant reduction of *Tak1* mRNA in the peri-infarct area (*p* = 0.045). A similar pattern was evident in the expression of *Nlrp3* and *Nlrc4*. Again, EPO reduced the expression of both inflammasomes in both genotypes (Fig. 2B, C). *Tak1*^*fl/fl*^ mice exhibited significantly higher mRNA levels of *Nlrp3* (*p* < 0.01) and *Nlrc4* (p < 0.01) than the *Cx3cr1*^*creER*^*-Tak1*^*fl/fl*^ mice 72 h after tMCAo. In contrast, mRNA levels of *Aim2* and of the adaptor protein *Asc* revealed no genotype-specific differences (Fig. 2D, E). The transcript levels of *Casp1* were significantly reduced after deletion of Mi/MΦ TAK1 both after 6 (*p* < 0.05) as well as after 72 h (*p* < 0.05) of reperfusion. In addition, EPO significantly reduced mRNA levels of *Casp1* in both genotypes and at both reperfusion times (*p* < 0.05) (Fig. 2F). Similarly, EPO also reduced post-ischemic upregulated mRNA levels of IL-1β at both reperfusion timepoints, while the other cleavage product of inflammasomes, IL-18, did not appear to be affected by either ischemia or EPO administration (Fig. 2H).

This data shows that cerebral ischemia among other priming stimuli, such as ligands for toll-like receptors (TLRs), NLRs (e.g., NOD1 and NOD2), or cytokine receptors, is able to upregulate the transcription of inflammasomes and *pro-Il-1β*. Furthermore, EPO appears to reduce the priming signal of inflammasomes.

### EPO Dampened Stroke-Induced Activation of TAK1 and Inflammasome Cascades

**Fig. 3:**
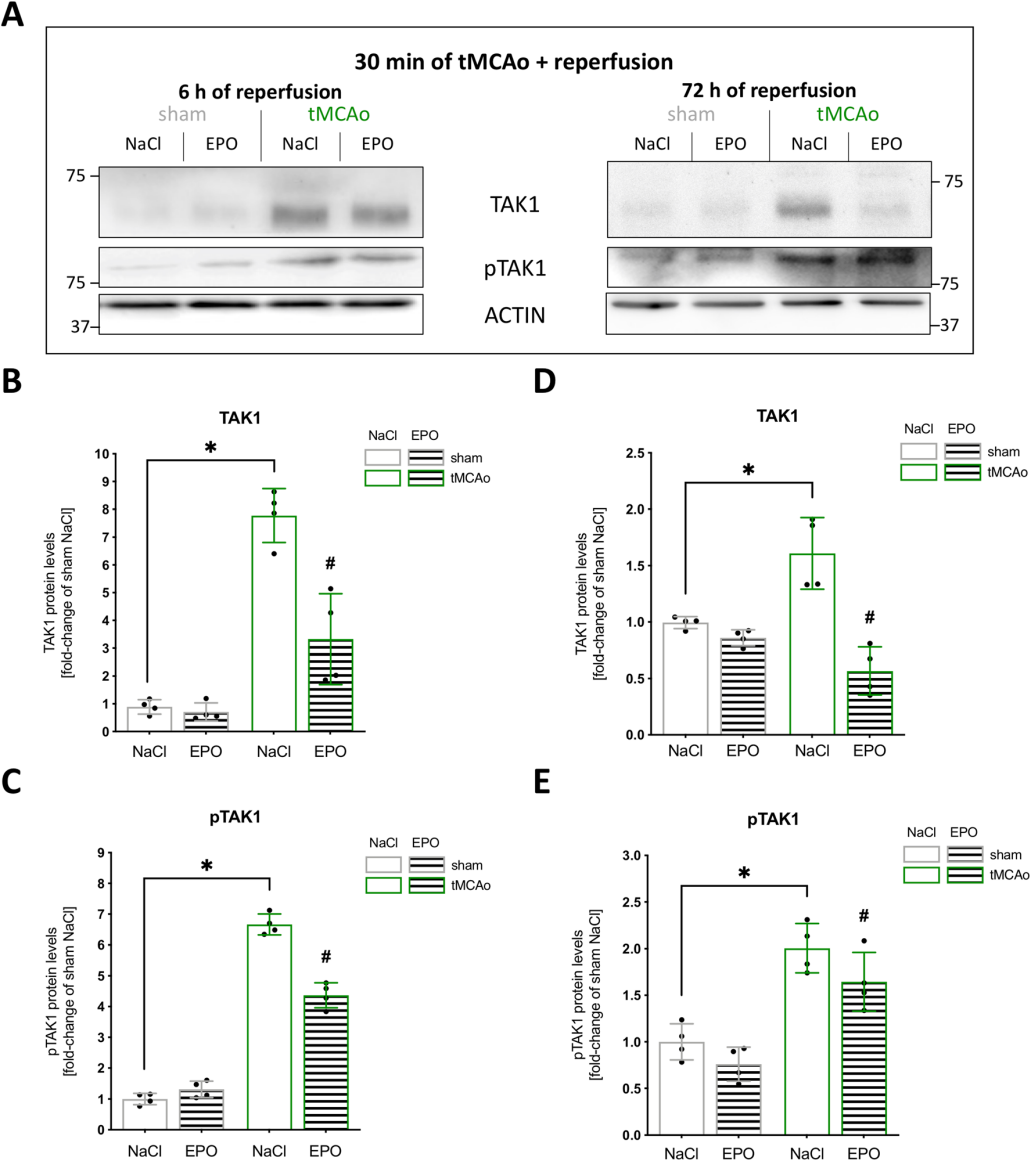
Ischemia-induced upregulation and activation of TAK1 were dampened by EPO. **A** Representative immunoblotting images of TAK1, pTAK1, ACTIN after 30 min sham or tMCAo surgery with 6- or 72-h reperfusion. **B–E** Protein levels of TAK1 and pTAK1 in peri-infarct zone were detected by immunoblotting (*n* = 4). ACTIN served as loading control. All bars represent means ± SD, individual data points are shown. The time points were blotted separately and normalized to their corresponding NaCl control. For statistical analysis, a 3-way ANOVA followed by Tukey’s multiple comparisons test was performed. Comparison of each group at different time-points: #*p* < 0.05 EPO treatment compared to the NaCl group, *p < 0.05 for intergroup comparison

To further assess the influence of cerebral ischemia on the activation of TAK1 and the inflammasomes NLRP3, NLRC4, and AIM2, we first compared the protein expression of TAK1 and its phosphorylation (pTAK1) in mice after tMCAo or sham surgery (Fig. 3A). The post-ischemic brain biopsies from the periinfarct zone exhibited a significant increase of TAK1 protein levels after a reperfusion time of 6 h compared to the sham group (Fig. 3B) (*p* = <0.0001). A similar pattern was observed in the protein levels of pTAK1 (Fig. 3C) (*p* = <0.0001). However, after 72 h of reperfusion, the stroke group still presented with higher levels of TAK1 and pTAK1 compared to the sham group, though the difference was not as prominent as at the earlier reperfusion timepoint (6 h) (Fig. 3D, E). EPO administration resulted in a significant reduction of post-ischemic elevated protein levels of both TAK1 and pTAK1 at both reperfusion times (*p* < 0.05).

**Fig. 4:**
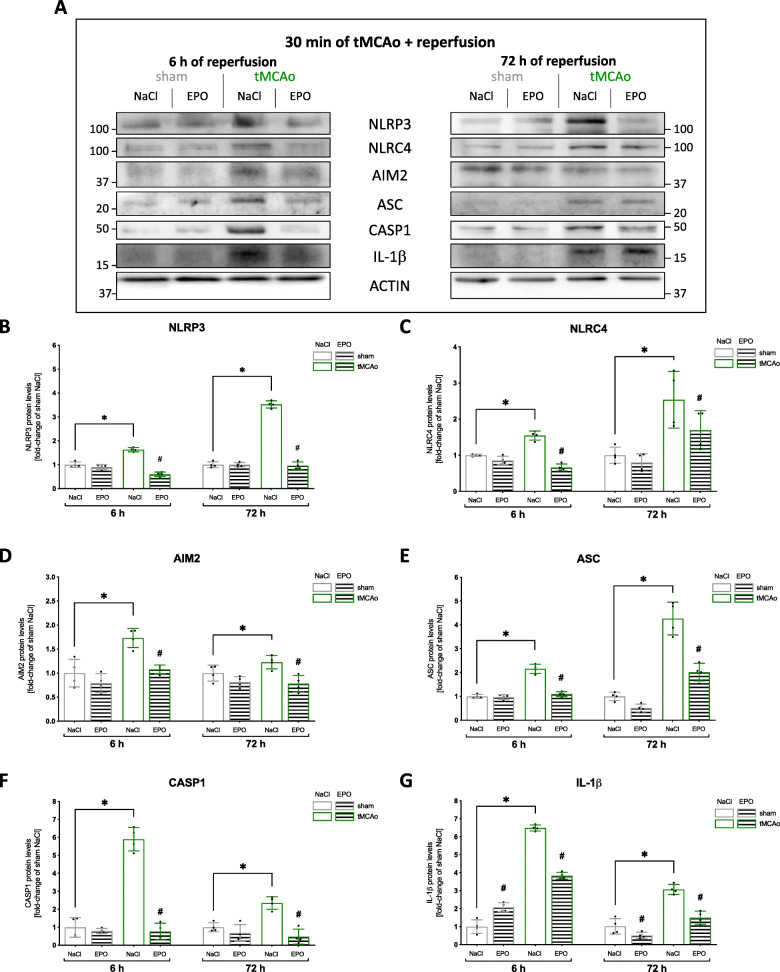
EPO administration abrogated the activation of the NLRP3, NLRC4, and AIM2 inflammasomes after stroke. **A** Representative immunoblotting images of NLRP3, NLRC4, AIM2, ASC, CAPS1, IL-1β and ACTIN after 30 min sham or tMCAo surgery with 6 or 72 h reperfusion **B–G** Protein-levels of NLRP3, NLRC4, AIM2, ASC, CAPS1, IL-1β in peri-infarct zone were detected by immunoblotting (*n* = 4). ACTIN served as loading control. Quantification of protein levels by densitometric analysis. All bars represent means ± SD, individual data points are shown. The time points were blotted separately and normalized to their corresponding NaCl control. For statistical analysis, a 3way ANOVA followed by Tukey’s multiple comparisons test was performed. Comparison of each group at different time-points: #*p* < 0.05 EPO treatment compared to the NaCl group, *p < 0.05 for intergroup comparison

Next, we investigated the influence of stroke and EPO-administration on the activation of the inflammasomes NLRP3, NLRC4 and AIM2, and their downstreaming cascade (Fig. 4A). We found higher protein levels of all three inflammasomes after stroke compared to the sham group (*p* < 0.05) at both reperfusion timepoints (Fig. 4B–D). However, while the protein levels of NLRP3 and NLRC4 increased in the course of the reperfusion period, AIM2 showed the opposite trend (Fig. 4D). EPO reduced the stroke-induced elevated protein levels of all three inflammasomes at both reperfusion timepoints (*p* < 0.05). Cerebral ischemia also resulted in a significant increase of protein levels of the adaptor protein ASC (Fig. 4E), activated Caspase-1 (Fig. 4F), and the cleavage product of inflammasomes, IL-1β (Fig. 4G) (*p* < 0.05). Again, EPO was able to significantly reduce the protein levels of the aforementioned proteins in both reperfusion phases.

In conclusion, in its acute phase, cerebral ischemia not only leads to increased expression but also to enhanced activation of TAK1 and the inflammasome complexes of NLRP3, NLRC4, and AIM2. Both of these effects were suppressed after EPO administration, providing further insight into the anti-apoptotic/pyroptotic and anti-inflammatory effects of EPO after ischemic stroke.

### Post-Ischemic Upregulation and Activation of the NLRP3, NLRC4, and AIM2 Inflammasomes Appeared to Depend on Mi/MΦ TAK1

**Fig. 5:**
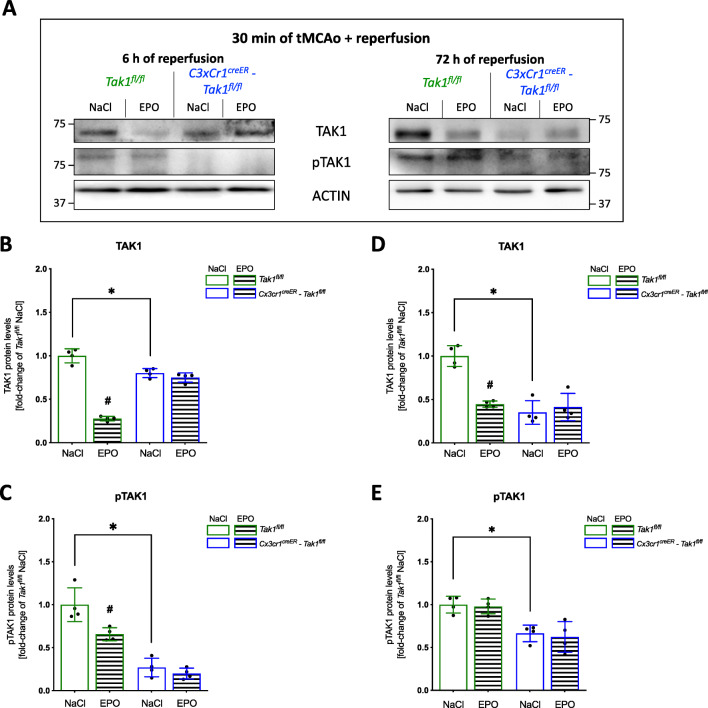
Microglial cells and macrophages appeared to be the main source of TAK1 after stroke**. A** Representative immunoblotting images of TAK1, pTAK1, ACTIN after 30 min of tMCAo surgery with 6 or 72 h reperfusion. **B–E** Protein levels of TAK1 and pTAK1 in peri-infarct zone were detected by immunoblotting (*n* = 4). ACTIN served as loading control. Quantification of protein levels by densitometric analysis. All bars represent means ± SD, individual data points are shown. The time points were blotted separately and normalized to their corresponding NaCl control. For statistical analysis, a 3-way ANOVA followed by Tukey’s multiple comparisons test was performed. Comparison of each group at different time-points #*p* < 0.05 EPO treatment compared to the NaCl group, *p < 0.05 for intergroup comparison

**Fig. 6:**
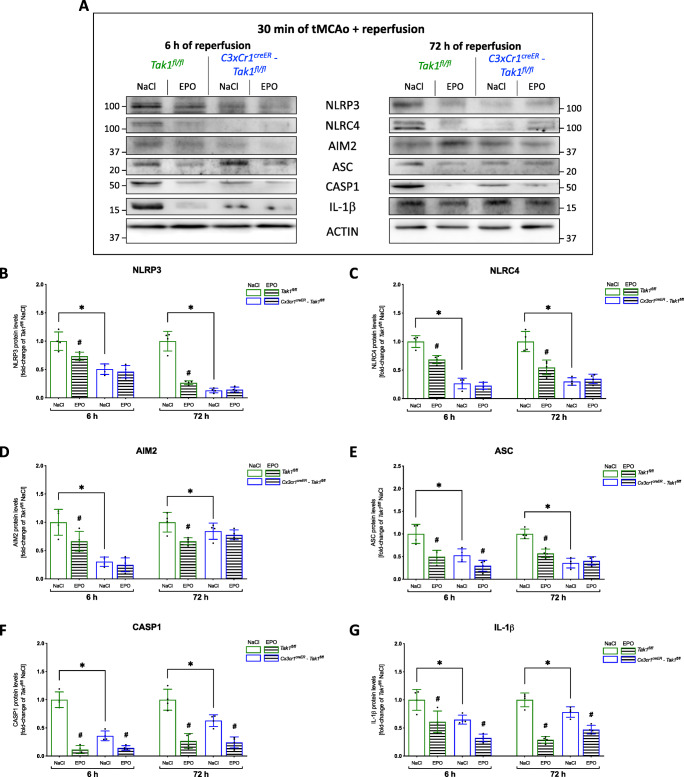
EPO-dependent regulation of the inflammasomes is abolished after deletion of Mi/MΦ TAK1. **A** Representative immunoblotting images of NLRP3, NLRC4, AIM2, ASC, CAPS1, IL-1β, and ACTIN after 30 min tMCAo surgery with 6 or 72 h reperfusion. **B–G** Protein-levels of NLRP3, NLRC4, AIM2, ASC, CAPS1, IL-1β in peri-infarct zone 6 or 72 h after tMCAO were detected by immunoblotting (*n* = 4). ACTIN served as loading control. Quantification of protein levels by densitometric analysis. All bars represent means ± SD, individual data points are shown. The time points were blotted separately and normalized to their corresponding NaCl control. For statistical analysis, a 3-way ANOVA followed by Tukey’s multiple comparisons test was performed. Comparison of each group at different time-points #*p* < 0.05 EPO treatment compared to the NaCl group, *p < 0.05 for intergroup comparison

To investigate a possible association between TAK1 and the observed post-ischemic upregulation of the NLRP3, NLRC4, and AIM2 inflammasomes, we utilized biopsies from the peri-infarct zones of *Tak1*^*fl/fl*^ and *Cx3cr1*^*creER*^*-Tak1*^*fl/fl*^ mice for protein analysis. We demonstrated that TAK1 was present in lower abundance after both 6 and 72 h of reperfusion after deletion of Mi/MΦ TAK1 than in the *Tak1*^*fl/fl*^ control mice (*p* < 0.05) (Fig. 5A, B, D). This difference between the two genotypes was also evident in the protein levels of pTAK1 at both timepoints of reperfusion (Fig. 5C, E). EPO reduced protein levels of TAK1 and pTAK1 in the controls but not in the mice with deleted Mi/MΦ TAK1 (*p* < 0.05). In the latter, a significant reduction of protein levels of NLRP3, NLRC4, and AIM2 (*p* < 0.05) was observed compared to controls at both reperfusion periods (Fig. 6A–D). However, the most prominent difference in the levels of the abovementioned proteins between the two genotypes was particularly prominent at the 72 h reperfusion time. A marked difference between the two genotypes was also found in the levels of ASC, CASP1, and IL-1β (Fig. 6E–G). Here, we also observed a substantial reduction in the protein levels of ASC, CASP1, and IL-1β after the deletion of Mi/MΦ TAK1. EPO administration regulated protein levels from the inflammasomes at both reperfusion times, but not in animals with deletion of Mi/MΦ TAK1. However, EPO reduced protein levels of ASC, Caspase-1, and IL-1β (*p* < 0.05) in both genotypes and at both times of reperfusion.

In conclusion, our data suggest that Mi/MΦ appear to be a major source of TAK1 and pTAK1 expression after stroke. Mi/MΦ TAK1 seems to play an eminent role in the regulation and activation of NLRP3, NLRC4, and AIM2 inflammasomes. Furthermore, the regulatory effect of EPO on inflammasomes appears to depend in part on Mi/MΦ TAK1.

### NLRC4 and AIM2 Were Upregulated upon Functional Inhibition of the NLRP3 Inflammasome after Ischemia

**Fig. 7:**
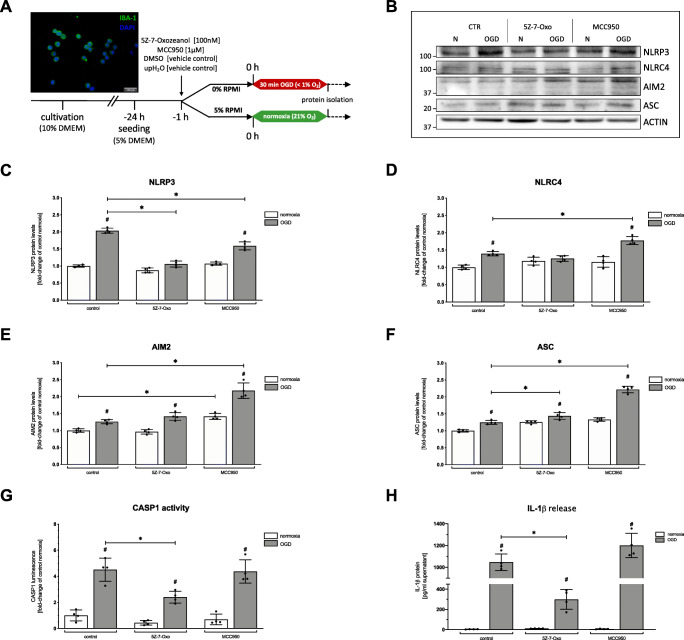
Impact of pharmacological inhibition of TAK1 and the NLRP3 inflammasome on microglial BV-2 cells after in vitro ischemia. **A** Schematic illustration of cell cultivation, treatment protocol, and representative images of IBA-1 and DAPI in microglial BV-2 cells. **B** Representative immunoblotting images of NLRP3, NLRC4, AIM2, ASC, and ACTIN after 90 min of OGD and normoxia. **C–F** Protein levels of NLRP3, NLRC4, AIM2, and ASC in cell lysate were detected by immunoblotting. ACTIN served as loading control. Quantification of protein levels by densitometric analysis. **G** Evaluation of Caspase-1 activity by Glow assay shown as relative luminescence to normoxia control **H** Concentration of IL-1β was detected by ELISA given in pg/mL supernatant. All bars represent means ± SD, individual data points are shown (*n* = 4). For statistical analysis, a 2-way ANOVA followed by Tukey’s multiple comparisons test was performed. Comparison of OGD for each treatment group #*p* < 0.05 compared to the corresponding normoxia, *p < 0.05 for intergroup comparison

To elucidate whether NLRP3 is the main inflammasome responsible for post-ischemic maturation of IL-1β in microglial cells, we used MCC950 to functionally inhibit NLRP3 (Fig. 7A). For the inhibition of TAK1 in microglial BV-2 cells, we utilized 5Z-7-Oxozeaenol. Increased HIF-1α protein levels assured a sufficient hypoxic stimulus (online resource 4A, B). OGD (O_2_ < 1%) followed by different reperfusion timepoints revealed an increase in cell death, which was abrogated by 5Z-7-Oxozeaenol, but not upon MCC950 treatment (online resource 3E-G). IL-1β concentrations were significantly increased directly after OGD (0 h of reperfusion), which declined considerably after 6 h of reperfusion (online resource 3F). In line with our in vivo findings, protein levels of TAK1 and pTAK1 were elevated after OGD (online resource 4A,C,D).

Western blot analysis revealed a significant up-regulation of all three inflammasomes (NLRP3, NLRC4, and AIM2) as well as the adapter protein ASC after OGD (*p* < 0.03) (Fig. 7B–F). Similarly, CASP1 and IL-1β were also significantly increased after OGD (*p* < 0.001) (Fig. 7G, H).

Administration of 5Z-7-Oxozeaenol diminished the OGD dependent increase of NLRP3 and NLRC4 but not of AIM2 protein levels. However, CASP1 activity as well as the amount of IL-1β were significantly reduced upon 5Z-7-Oxozeaenol after OGD treatment (*p* < 0.02). MCC950 administration mitigated the OGD-induced upregulation of NLRP3, but increased protein levels of NLRC4, AIM2, and ASC (*p* < 0.001). CASP1 activity and IL-1β levels showed no significant difference compared to the control condition (Fig. 7C–H).

In conclusion, our in vitro data suggest that microglial TAK1 impacts the protein levels and activation of NLRP3, NLRC4 and AIM2 after OGD. The reduction of CASP1 activity and IL-1β protein levels upon application of 5Z-7-Oxozeaenol indicates a major role of TAK1 in the microglial maturation of IL-1β. The NLRC4 and AIM2 inflammasomes appear to functionally compensate for the inhibition of NLRP3 regarding the maturation of IL-1β.

## Discussion

Decades of preclinical and clinical research provide evidence that neuroinflammation in the acute phase of stroke is detrimental and thus considered a prime target for the development of neuroprotective therapies [52]. In this context, addressing frequently observed cytokines (TNF-α, IL-1β, IL-6), or particularly their upstream regulators such as TAK1, holds much promise. Microglia, the main immunocompetent and macrophage-like cells of the central nervous system (CNS), are a major source of TAK1 expression [20]. Several studies have demonstrated an upregulation and activation of TAK1 accompanied by a plethora of cytokines and chemokines in different disease models [18, 20, 53, 54]. In line with our previous study, we here demonstrated that 30 min of tMCAo followed by both 6 and 72 h of reperfusion significantly increased the protein levels and activation of TAK1. The same was true for the cytokines TNF-α, IL-6, and IL1-β [20]. The reperfusion time points addressed in this study were chosen to investigate the anti-inflammatory capacities of EPO in the acute and subacute phase after ischemic stroke and to allow potential comparisons with our previous data and several (pre)clinical studies [20, 55]. Since little to nothing is known about the impact of EPO on microglia, microglial TAK1, and on the inflammasomes after stroke, we deemed an investigation of the reperfusion time point of 6 h after 30 min of tMCAo essential to draw any conclusion about a potential EPO/TAK1/inflammasome axis. The subacute reperfusion phase of 72 h after stroke was chosen on the basis of our preliminary data, which showed a significant reduction in infarct volumes after this period upon three administrations of EPO. In the acute post-ischemic/OGD phase, TAK1 depletion/inhibition markedly improved neurobehavioral outcomes and decreased cell death as well as inflammation (Figs. 1, 6, and 7, and online resource 3). However, similar to microglial cells, TAK1 also seems to possess a double-edged sword function depending on the stimulus and the cell type [21, 56, 57]. While some studies attributed TAK1 pro-apoptotic and pro-inflammatory effects in mouse embryonic fibroblasts (MEFs) and keratinocytes, others reported its crucial role in restricting cell death and mediating cell growth in neutrophils and macrophages [35, 54, 58]. In stroke models, TAK1 has so far been shown to exert pro-apoptotic and pro-inflammatory effects and thereby inhibition of TAK1 has been implicated in cytoprotection. Chauhan and colleagues demonstrated that the depletion of myeloid-specific TAK1 resulted in reduced brain monocyte infiltration and improved outcomes after stroke [24]. We have shown in both our previous and current studies that deletion of Mi/MΦ TAK1 leads to a significant reduction in inflammation and cell death. We also observed similar effects upon general pharmacological inhibition of TAK1. Although other brain-resident cells such as astrocytes, CNS-associated macrophages, and mast cells also orchestrate post-ischemic neuroinflammation [59–62], besides others, our previous as well as recent study indicate that microglia and in particular Mi/MΦ TAK1 are primarily in charge of post-ischemic inflammation [20, 63]. Furthermore, we provided evidence that the deletion of Mi/MΦ TAK1 results in more sustained protection compared to general pharmacological inhibition highlighting the cell-specific properties of TAK1 [20]. Our model represents a conditional monocytic TAK1 depletion and does not induce an ubiquitous knockout of TAK1. Since the protein analyses were performed on biopsies along the peri-infarct zone and not in single-cell lysates, the influence of cells other than microglia and monocytes should not be neglected. TAK1 is also expressed by other brain resident neuroglial cells, which may have upregulated TAK1 in the high acute phase after tMCAo and thus could explain the relatively high TAK1 levels also in Cx3cr1^creER-^TAK1^fl/fl^ mice. Neuroinflammation, in particular, post-stroke levels of IL-1β have been demonstrated to be downregulated after TAK1 inhibition in different stroke models [20, 64, 65]. However, the precise mechanism regarding the expression and maturation of IL-1β remains to be elucidated.

The maturation of IL-1β depends on inflammasomes, among which NLRP3-containing inflammasome is the best-studied inflammasome in the scope of stroke. A recent literature search on NCBI (15th January 2021) using the search terms “NLRP3,” “NLRC4,” and “AIM2” in combination with “stroke,” “tMCAo,” and “cerebral ischemia” revealed 15–25 times the number of studies for NLRP3 (263) than for NLRC4 (10) and AIM2 (15). TAK1 is reported to regulate the activation of the NLRP3 inflammasome [35, 59, 66, 67] but little is known about the influence of TAK1 on NLRP3, NLRC4, and AIM2 inflammasomes after stroke. We have shown that after both tMCAo and OGD, protein levels and activation of TAK1 and the aforementioned inflammasomes are significantly increased (Figs. 3, 4, and 7, and online resource 4). Concomitantly, we provide evidence that microglial cells are a major source of inflammasomes after acute stroke (online resource 2) and that TAK1 regulates NLRC4 and AIM2 in addition to NLRP3. After Mi/MΦ TAK1 deletion, we found markedly reduced protein levels of the three inflammasomes accompanied by a decrease of both Caspase-1 activation and IL-1β maturation (Fig. 6). Conversely, Franke and colleagues have most recently reported that NLRP3 is mainly expressed in post-ischemic murine neurons after 60 min of tMCAo followed by a reperfusion period of 24 h [68]. Analyzing bulk biopsies from the peri-infarct zone, we demonstrated that deletion of Mi/MΦ TAK1 significantly reduced the expression and activation of the three inflammasomes after both hyperacute (6 h) as well as subacute (72 h) reperfusion periods following stroke (Fig. 6). Although this does not exclude the expression of the abovementioned inflammasomes in the other CNS cells, it nevertheless indicates their predominant microglial expression. Whether microglial NLRP3 is the major player in the post-ischemic activation of Caspase-1 and of IL-1β has not been investigated so far. Cao et al. have recently shown that deletion of *Nlrp3* had no impact on the regulation of IL-1β after LPS stimulation [44]. In line with this, we found no effect on post-hypoxic activation of Caspase-1 and IL-1β in microglial BV-2 cells after functional inhibition of NLRP3 using MCC950. Pharmacological inhibition of TAK1, on the other hand, led to a significant reduction of the inflammasomes NLRP3, NLRC4, and AIM2 as well as to a decrease in the activation of Caspase-1 and IL-1β (Fig. 7). The observed upregulation of NLRC4 and AIM2 following MCC950 treatment may compensate for the functional inhibition of NLRP3 regarding the maturation of IL-1β. Whether TAK1 might also have a regulatory effect on other members of the inflammasome family, or even potentially act as an upstream activator of all known inflammasomes, cannot be ruled out and is certainly worth investigating in future studies.

Using C-X3-C motif chemokine receptor 1 (Cx3cr1), which is also expressed e.g. by macrophages, as a promoter for the conditional knockout of TAK1, raises the question to what extent the protective effects are indeed mediated only by microglial cells. However, it has previously been shown that this probability might be rather small due to differences in the turnover rates. In fact, while microglial cells lack TAK1 at 4 weeks after tamoxifen administration, other macrophage-like cells (macrophages, dendritic cells) exhibit normal TAK1 levels again after this period [18, 20, 22]. We have previously indicated that CNS-associated macrophages (CAMs) are a brain resident stable cell population demonstrating no substantial exchanges with peripheral blood cells [69]. However, the proportion of CAMs relative to microglial cell has not been accurately evaluated to date, so we cannot attribute the observed effects precisely to one cell type. Most recently Masuda et al. have introduced *Hexb* as a stable microglial core gene [70]. Future studies should consider *Hexb* as a microglial-specific promotor to avoid co-tracing of other monocytic cells.

A large number of studies have already shown that the pleiotropic and anti-inflammatory cytokine EPO has a regulatory impact on the downstream effectors of TAK1 (e.g., p38MAPK, NF-κB, IL-6, TNF-α) as well as on IL-1β, IL-18, and the inflammasome NLRP3 [43, 44, 71, 72]. However, a direct interaction between EPO, TAK1, and inflammasomes, especially after stroke, has not been investigated to date. In line with our previous studies [37, 45, 73], EPO reduced infarct sizes, neurological impairments, and attenuated post-stroke weight loss over the observation period of 72 h. These effects upon EPO administration were further enhanced in combination with Mi/MΦ TAK1 deletion (Fig. 1). The suppressive effect of EPO on TAK1/inflammasomes in the other cells should not be neglected and could possibly explain the protective effects in the Cx3cr1^creER^-Tak1^fl/fl^ mice. Furthermore, we demonstrated that EPO dampened stroke-induced activation of TAK1 and the inflammasomes along with their downstream cascade. However, the reduction of the protein levels of the inflammasomes was not evident after the deletion of Mi/MΦ TAK1, indicating that EPO-dependent regulation of the inflammasomes might be conveyed through TAK1. It is debatable whether the protein levels of inflammasomes in the Cx3cr1^creER^ TAK1^fl/fl^ mice are reduced by the knockout to the point that further reduction by EPO is no longer apparent or significant. However, since EPO administration downregulated the downstreams (CASP1, IL-1β), it might be possible that EPO via TAK1 only has a regulatory effect on inflammasome sensors, not on the downstreams.

There is little literature on the regulation of TAK1 gene expression by EPO in the brain, however, NF-κB may play a crucial role here. Takahashi and colleagues identified a potential NF-κB -binding sequence in the promoter region of TAK1 in a colon cancer cell model [74]. Upon binding of the phosphorylated/activated NF-κB p65 to the TAK1 promoter region, TAK1 transcription appeared to be upregulated [74]. This would suggest a positive regulatory loop in which activation of TAK1 induces proinflammatory NF-κB signaling and NF-κB in turn leads to activation/upregulation of TAK1-transcription. Binding of EPO to the EPO receptor (EPOR) activates among others EPOR/JAK2/STAT3 signaling, thereby suppressing NF-κB p65 phosphorylation and transcriptional activity [44]. We thus hypothesize that the EPO-mediated regulation of TAK1 gene expression in the brain might proceed via NF-κB. However, this potential association should be investigated in future studies utilizing successive and selective inhibition of the TAK1/NF-κB p65 cascade in microglia after EPO administration. The EPO-conveyed inhibition of NF-κB p65 transcriptional activity further attenuates the expression of NLRP3, among others. This suggests that the EPO-mediated regulation of inflammasome complexes is not mediated mainly via TAK1 but is also conveyed by its inhibitory effect on NF-κB and thereby on the transcription and priming of inflammasome sensors and IL-1β [44, 74].

EPO has been clinically approved for the treatment of renal anemia for many years and has shown solid safety and efficacy after stroke in several preclinical studies [43, 44, 75, 76], thus, it was also tested in stroke patients. However, after a successful pilot study, EPO failed to show beneficial effects in a multicenter study. A subsequent subgroup analysis revealed that the combination of EPO with intravenous injections of recombinant tissue plasminogen activator (rtPA) caused complications such as bleedings and brain edema, whereas EPO administration on its own was protective [77]. In addition to thrombolysis with rtPA, endovascular stroke treatment (e.g., mechanical thrombectomy (MT)) is now FDA-approved and enables us to perform physical removal of the clot occluding a blood vessel of the brain within 24 h of stroke onset [78–80]. Considering that tMCAo strongly reflects reperfusion after mechanical thrombectomy, the translation of promising drugs such as EPO into the clinic as an adjunct therapy to MT might be successful [81, 82].

In conclusion, we demonstrated that a deletion of Mi/MΦ TAK1 as well as the administration of EPO reduced infarct sizes and neurological impairments after tMCAo in mice. In addition, we address major knowledge gaps related to EPO-dependent regulation of the NLRP3, NLRC4, and AIM2 inflammasomes after acute ischemic stroke. We provide evidence that microglia/CAMs and in particular their TAK1 are main contributors to post-ischemic neuroinflammation. Furthermore, we demonstrated that TAK1 regulates the expression and activation of the NLRP3, NLRC4, and AIM2 inflammasomes (Graphical abstract). Moreover, functional inhibition of NLRP3 is compensated by an upregulation of NLRC4 and AIM2 after ischemia. In addition, we demonstrated that EPO mitigated stroke-induced activation of TAK1 and the inflammasomes along with their downstream cascade, which was not evident after Mi/MΦ TAK1 deletion. In conclusion, our data indicate that monocytic TAK1 is crucially involved in the regulation of the inflammasomes in the brain after stroke and that EPO conveyed neuroprotection after stroke might be mediated via an EPO/TAK1/inflammasome axis.

## Supplementary Information

Online Resource 1 **Intraoperative laser doppler flowmetry revealed no differences between genotypes. (A)** Schematic illustration of the treatment protocol for both genotypes, 4HT injections and EPO as well as NaCl treatment. **(B)** Scheme of protocol summarizing the number of total animals (64 mice). Exclusion criteria are described in material and methods section. **(C)** Changes in ipsilateral Laser Doppler flowmetry by intraluminal during MCAO procedure were monitored. The baseline blood flow was considered as 100% for all mice. Changes in LDF after occlusion of CCA, MCA and reperfusion are demonstrated. Abbreviations: bp: base pairs*;* CCAo: Common Carotid Artery occlusion; MCAo: Middle Cerebral Artery occlusion; LDF: Laser Doppler flowmetry. **Online Resource 2. Immunohistochemical analysis of the NLRP3, NLRC4 and AIM2 inflammasomes in microglial cells, astrocytes and neurons after acute ischemic stroke.** Immunofluorescence of brain slices after 30 min of tMCAo and 72 h of reperfusion in Tak1fl/fl and Cx3cr1creER –Tak1fl/fl treated with NaCl or EPO. (A + E) Representative images of double staining of a inflammasome such as NLRP3, NLRC4 or AIM2 (green) and a cell marker like NeuN, GFAP or Iba1 (red); nuclei were counterstained with DAPI (blue). (B-D + F-H) Double positives cells per 100 cells in the periinfarct zone are given in percentage. Data are presented as mean ± SD. Differences were tested by Mann-Whitney (*n* = 4). A list of used Antibodies is given in Table 3. **Online Resource 3. In vitro stroke model, cell viability and IL-1β protein levels in murine microglial cells. (A)** Schematic illustration of cell cultivation and treatment protocol of BV-2 cells for viability assessment. **(B)** Environmental conditions during hypoxia in the chamber and the supernatant. **(C + D)** LDH release in supernatant in % of lysis control during normoxia and after OGD with different concentrations of 5Z-7-Oxozeaenol and MCC950. **(E)** LDH release in the supernatant in % of lysis control during normoxia and after OGD at different reperfusion timepoints (n = 4). **(F)** Viability in % of total cell count during normoxia and after OGD at different reperfusion timepoints. **(G)** LDH release in the supernatant in % of lysis control under CTR, 5Z-7-Oxozeaenol and MCC950 during normoxia, 0 and 6 h after OGD (n = 4). **(H)** Concentration of IL-1β was detected directly after OGD and after 6 h reperfusion by ELISA given in pg/ml supernatant. All bars represent means ± SD, individual data points are shown. For statistical analysis (C/D) a Kruskal-Wallis test followed by Dunn’s multiple comparisons test was performed. For statistical analysis (F) a 2way ANOVA followed by Tukey’s multiple comparisons test was performed. Comparison of OGD for each treatment group #*p* < 0.05 compared to the corresponding normoxia, *p < 0.05 for intergroup comparison. **Online Resource 4. HIF-1α, TAK1 and pTAK1 levels after OGD in murine microglial cells. (A)** Representative immunoblotting images of HIF-1α, TAK1, pTAK1 and ACTIN after 90 min of OGD and normoxia. **(B-D)** Protein-levels of HIF-1α, TAK1 and pTAK1 in cell lysate were detected by immunoblotting (n = 4). ACTIN served as loading control. Quantification of protein-levels by densiometric analysis. For statistical analysis a 2way ANOVA followed by Tukey’s multiple comparisons test was performed. Bars represent means ± SD. *p < 0.05. (PDF 3087 kb)

## Data Availability

The datasets generated during and/or analyzed during the current study are available from the corresponding author on reasonable request.
