# Correction to: Erythropoietin Abrogates Post-Ischemic Activation of the NLRP3, NLRC4, and AIM2 Inflammasomes in Microglia/Macrophages in a TAK1-Dependent Manner

**DOI:** 10.1007/s12975-023-01156-2

**Published:** 2023-06-22

**Authors:** Ole Heinisch, Thomas Zeyen, Tobias Goldmann, Marco Prinz, Michael Huber, Jennifer Jung, Eren Arik, Shahin Habib, Alexander Slowik, Arno Reich, Jörg B. Schulz, Pardes Habib

**Affiliations:** 1 Department of Neurology, Medical Faculty, RWTH Aachen University, Pauwelsstraße 30, D-52074 Aachen, Germany; 2 Department of Neurology, University Hospital of Bonn, Bonn, Germany; 3 Institute of Neuropathology, Faculty of Medicine, University of Freiburg, Freiburg, Germany; 4 Signalling Research Centres BIOSS and CIBSS, University of Freiburg, Freiburg, Germany; 5 Center for Basics in NeuroModulation (NeuroModulBasics), Faculty of Medicine, University of Freiburg, Freiburg, Germany; 6 Institute of Biochemistry and Molecular Immunology, Medical Faculty, RWTH Aachen University, Aachen, Germany; 7 Medical Biochemistry, Department of Biochemistry, University of Leicester, Leicester, UK; 8 Department of Anatomy and Cell Biology, Medical Faculty, RWTH Aachen University, Aachen, Germany; 9 JARA-BRAIN Institute of Molecular Neuroscience and Neuroimaging, Forschungszentrum Jülich GmbH and RWTH Aachen University, Aachen, Germany

**Correction to: Translational Stroke Research**

10.1007/s12975-021-00948-8

We would like to make a clarification in the Authors Contribution. We would like to change “writing” used in the author’s contribution to “correction and editing of revision” to differentiate the various stages of manuscript preparation. We have described “writing” in the actual sense with “preparation of original draft”: OH in the manuscript. The writing actually refers to the final submission after responding to all reviewer comments.

The original article has been corrected.

