# Supplementary material for: Erythropoietin Abrogates Post-Ischemic Activation of the NLRP3, NLRC4, and AIM2 Inflammasomes in Microglia/Macrophages in a TAK1-Dependent Manner
Source: Transl Stroke Res. 2021 Oct 9;13(3):462–82. doi: 10.1007/s12975-021-00948-8 (PMC9046144; doi:10.1007/s12975-021-00948-8)

Erythropoietin abrogates post-ischemic activation of the NLRP3, NLRC4 and AIM2 inflammasomes in microglia/macrophages in a TAK1-dependent manner

Ole Heinisch[1], Thomas Zeyen[1,2], Tobias Goldmann[3], Marco Prinz[3,4,5], Michael Huber[6], Jennifer Jung[1], Eren Arik[1], Shahin Habib[7], Alexander Slowik[8], Arno Reich[1], Jörg B. Schulz[1,9], Pardes Habib[1,6,9, CA]

Department of Neurology, Medical Faculty, RWTH Aachen University, Aachen, Germany, 2) Department of Neurology, University Hospital of Bonn, Bonn, Germany, 3) Institute of Neuropathology, Faculty of Medicine, University of Freiburg, Freiburg, Germany, 4) Signalling Research Centres BIOSS and CIBSS, University of Freiburg, Freiburg, Germany. 5) Center for Basics in NeuroModulation (NeuroModulBasics), Faculty of Medicine, University of Freiburg, Freiburg, Germany. 6) Institute of Biochemistry and Molecular Immunology, Medical Faculty, RWTH Aachen University, Aachen, Germany. 7) Medical Biochemistry, Department of Biochemistry, University of Leicester, Leicester, United Kingdom. 8) Department of Anatomy and Cell Biology, Medical Faculty, RWTH Aachen University, Aachen, Germany. 9) JARA-BRAIN Institute of Molecular Neuroscience and Neuroimaging, Forschungszentrum Jülich GmbH and RWTH Aachen University, Aachen, Germany
Corresponding author: Pardes Habib, M.D./Ph.D., Department of Neurology, RWTH Aachen University, Pauwelsstraße 30, D-52074 Aachen, Germany, Phone: +49 241 80 37836, Fax: +49 241 80 82582, Email: phabib@ukaachen.de

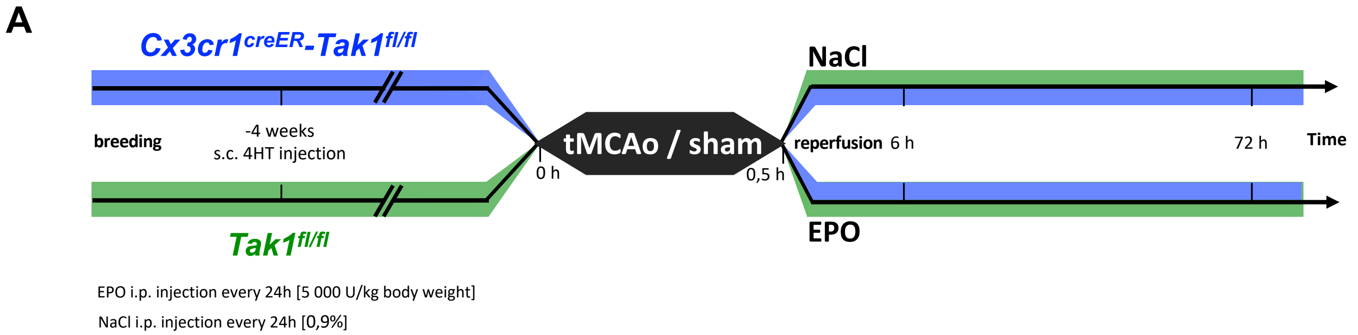

B

Model	sham				30 min tMACo							
Animal numbers	16				48							
Genotype	*Tak1fl/fl* / *Cx3cr1creER-Tak1fl/fl*				*Tak1fl/fl*				*Cx3cr1creER-Tak1fl/fl*			
Animal numbers	16				24				24			
Reperfusion time	6 h		72 h		6 h		72 h		6 h		72 h	
Animal numbers	8		8		12		12		12		12	
Treatment	NaCl	EPO	NaCl	EPO	NaCl	EPO	NaCl	EPO	NaCl	EPO	NaCl	EPO
Animal numbers	4	4	4	4	6	6	6	6	6	6	6	6

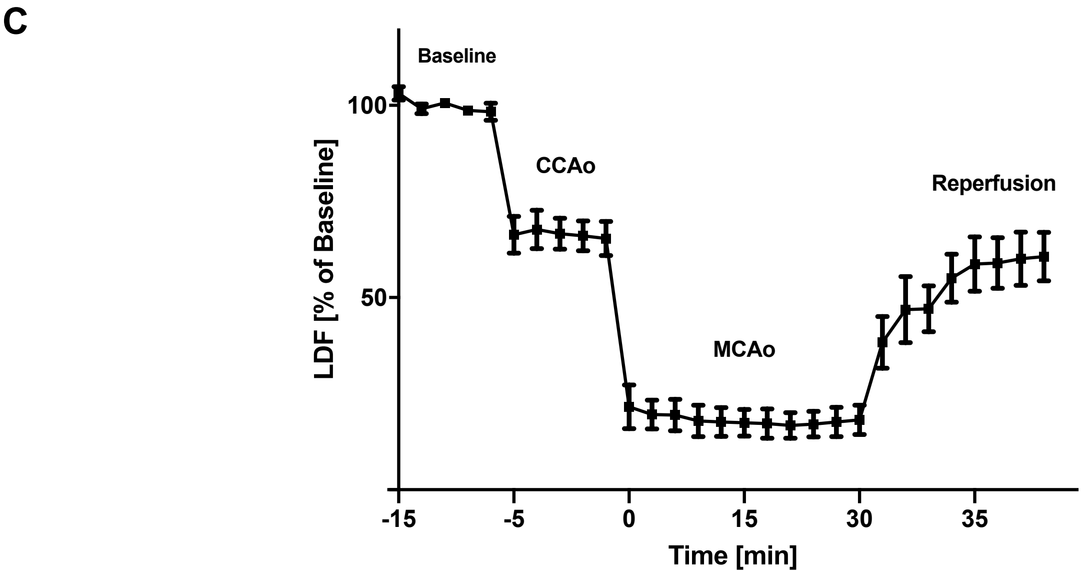

Erythropoietin abrogates post-ischemic activation of the NLRP3, NLRC4 and AIM2 inflammasomes in microglia/macrophages in a TAK1-dependent manner

Ole Heinisch[1], Thomas Zeyen[1,2], Tobias Goldmann[3], Marco Prinz[3,4,5], Michael Huber[6], Jennifer Jung[1], Eren Arik[1], Shahin Habib[7], Alexander Slowik[8], Arno Reich[1], Jörg B. Schulz[1,9], Pardes Habib[1,6,9, CA]

Department of Neurology, Medical Faculty, RWTH Aachen University, Aachen, Germany, 2) Department of Neurology, University Hospital of Bonn, Bonn, Germany, 3) Institute of Neuropathology, Faculty of Medicine, University of Freiburg, Freiburg, Germany, 4) Signalling Research Centres BIOSS and CIBSS, University of Freiburg, Freiburg, Germany. 5) Center for Basics in NeuroModulation (NeuroModulBasics), Faculty of Medicine, University of Freiburg, Freiburg, Germany. 6) Institute of Biochemistry and Molecular Immunology, Medical Faculty, RWTH Aachen University, Aachen, Germany. 7) Medical Biochemistry, Department of Biochemistry, University of Leicester, Leicester, United Kingdom. 8) Department of Anatomy and Cell Biology, Medical Faculty, RWTH Aachen University, Aachen, Germany. 9) JARA-BRAIN Institute of Molecular Neuroscience and Neuroimaging, Forschungszentrum Jülich GmbH and RWTH Aachen University, Aachen, Germany

Corresponding author: Pardes Habib, M.D./Ph.D., Department of Neurology, RWTH Aachen University, Pauwelsstraße 30, D-52074 Aachen, Germany, Phone: +49 241 80 37836, Fax: +49 241 80 82582, Email: phabib@ukaachen.de

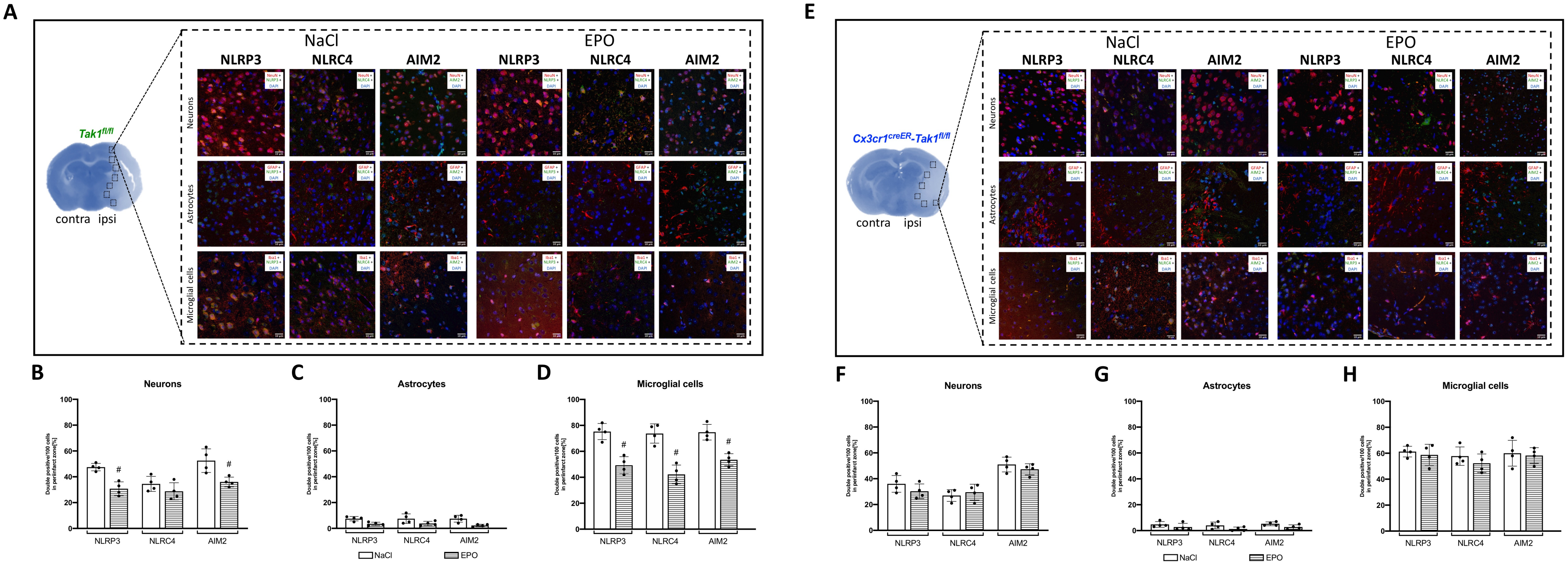

Erythropoietin abrogates post-ischemic activation of the NLRP3, NLRC4 and AIM2 inflammasomes in microglia/macrophages in a TAK1-dependent manner

Ole Heinisch[1], Thomas Zeyen[1,2], Tobias Goldmann[3], Marco Prinz[3,4,5], Michael Huber[6], Jennifer Jung[1], Eren Arik[1], Shahin Habib[7], Alexander Slowik[8], Arno Reich[1], Jörg B. Schulz[1,9], Pardes Habib[1,6,9, CA]

Department of Neurology, Medical Faculty, RWTH Aachen University, Aachen, Germany, 2) Department of Neurology, University Hospital of Bonn, Bonn, Germany, 3) Institute of Neuropathology, Faculty of Medicine, University of Freiburg, Freiburg, Germany, 4) Signalling Research Centres BIOSS and CIBSS, University of Freiburg, Freiburg, Germany. 5) Center for Basics in NeuroModulation (NeuroModulBasics), Faculty of Medicine, University of Freiburg, Freiburg, Germany. 6) Institute of Biochemistry and Molecular Immunology, Medical Faculty, RWTH Aachen University, Aachen, Germany. 7) Medical Biochemistry, Department of Biochemistry, University of Leicester, Leicester, United Kingdom. 8) Department of Anatomy and Cell Biology, Medical Faculty, RWTH Aachen University, Aachen, Germany. 9) JARA-BRAIN Institute of Molecular Neuroscience and Neuroimaging, Forschungszentrum Jülich GmbH and RWTH Aachen University, Aachen, Germany
Corresponding author: Pardes Habib, M.D./Ph.D., Department of Neurology, RWTH Aachen University, Pauwelsstraße 30, D-52074 Aachen, Germany, Phone: +49 241 80 37836, Fax: +49 241 80 82582, Email: phabib@ukaachen.de

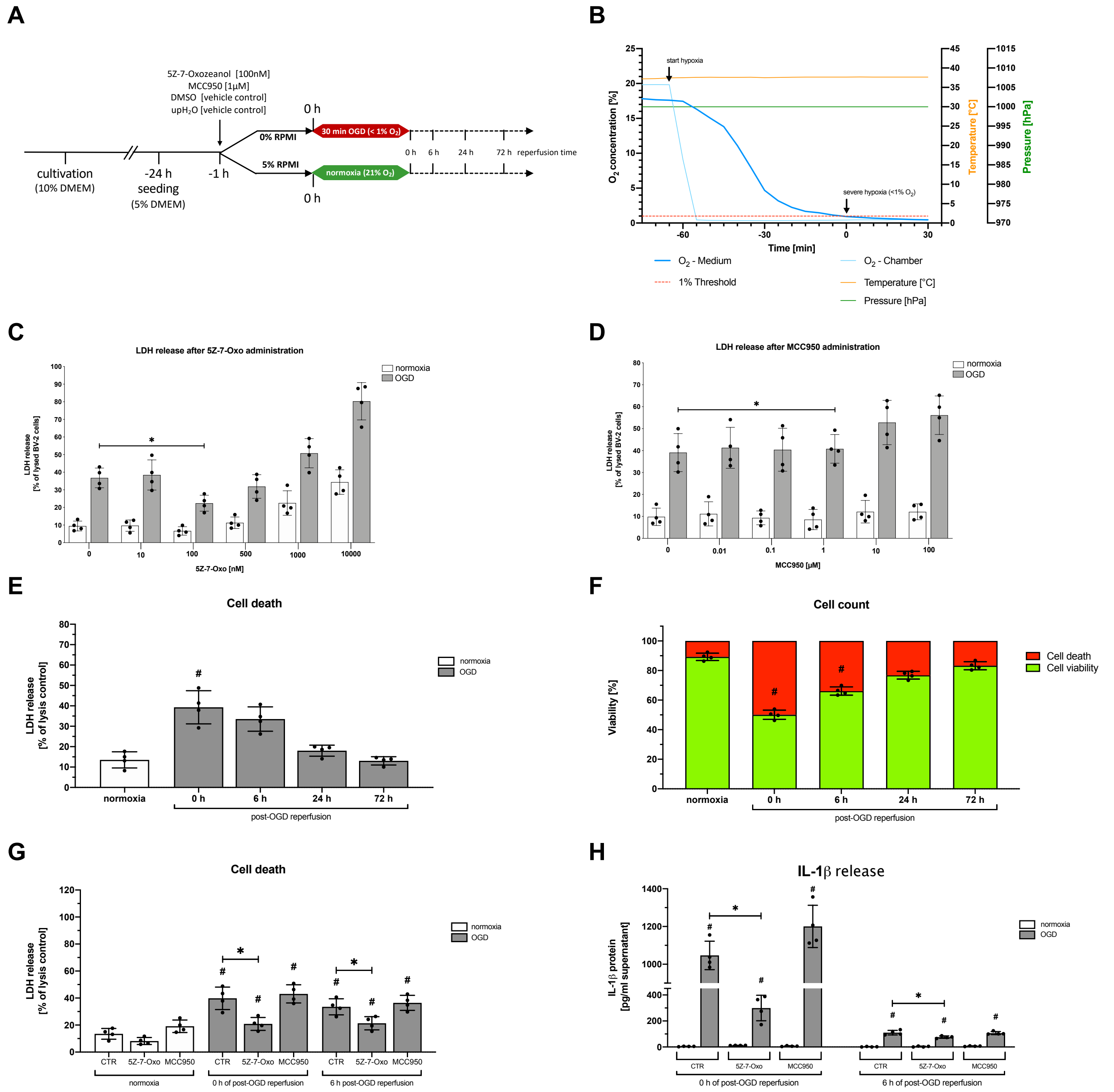

Erythropoietin abrogates post-ischemic activation of the NLRP3, NLRC4 and AIM2 inflammasomes in microglia/macrophages in a TAK1-dependent manner

Ole Heinisch[1], Thomas Zeyen[1,2], Tobias Goldmann[3], Marco Prinz[3,4,5], Michael Huber[6], Jennifer Jung[1], Eren Arik[1], Shahin Habib[7], Alexander Slowik[8], Arno Reich[1], Jörg B. Schulz[1,9], Pardes Habib[1,6,9, CA]

Department of Neurology, Medical Faculty, RWTH Aachen University, Aachen, Germany, 2) Department of Neurology, University Hospital of Bonn, Bonn, Germany, 3) Institute of Neuropathology, Faculty of Medicine, University of Freiburg, Freiburg, Germany, 4) Signalling Research Centres BIOSS and CIBSS, University of Freiburg, Freiburg, Germany. 5) Center for Basics in NeuroModulation (NeuroModulBasics), Faculty of Medicine, University of Freiburg, Freiburg, Germany. 6) Institute of Biochemistry and Molecular Immunology, Medical Faculty, RWTH Aachen University, Aachen, Germany. 7) Medical Biochemistry, Department of Biochemistry, University of Leicester, Leicester, United Kingdom. 8) Department of Anatomy and Cell Biology, Medical Faculty, RWTH Aachen University, Aachen, Germany. 9) JARA-BRAIN Institute of Molecular Neuroscience and Neuroimaging, Forschungszentrum Jülich GmbH and RWTH Aachen University, Aachen, Germany

Corresponding author: Pardes Habib, M.D./Ph.D., Department of Neurology, RWTH Aachen University, Pauwelsstraße 30, D-52074 Aachen, Germany, Phone: +49 241 80 37836, Fax: +49 241 80 82582, Email: phabib@ukaachen.de

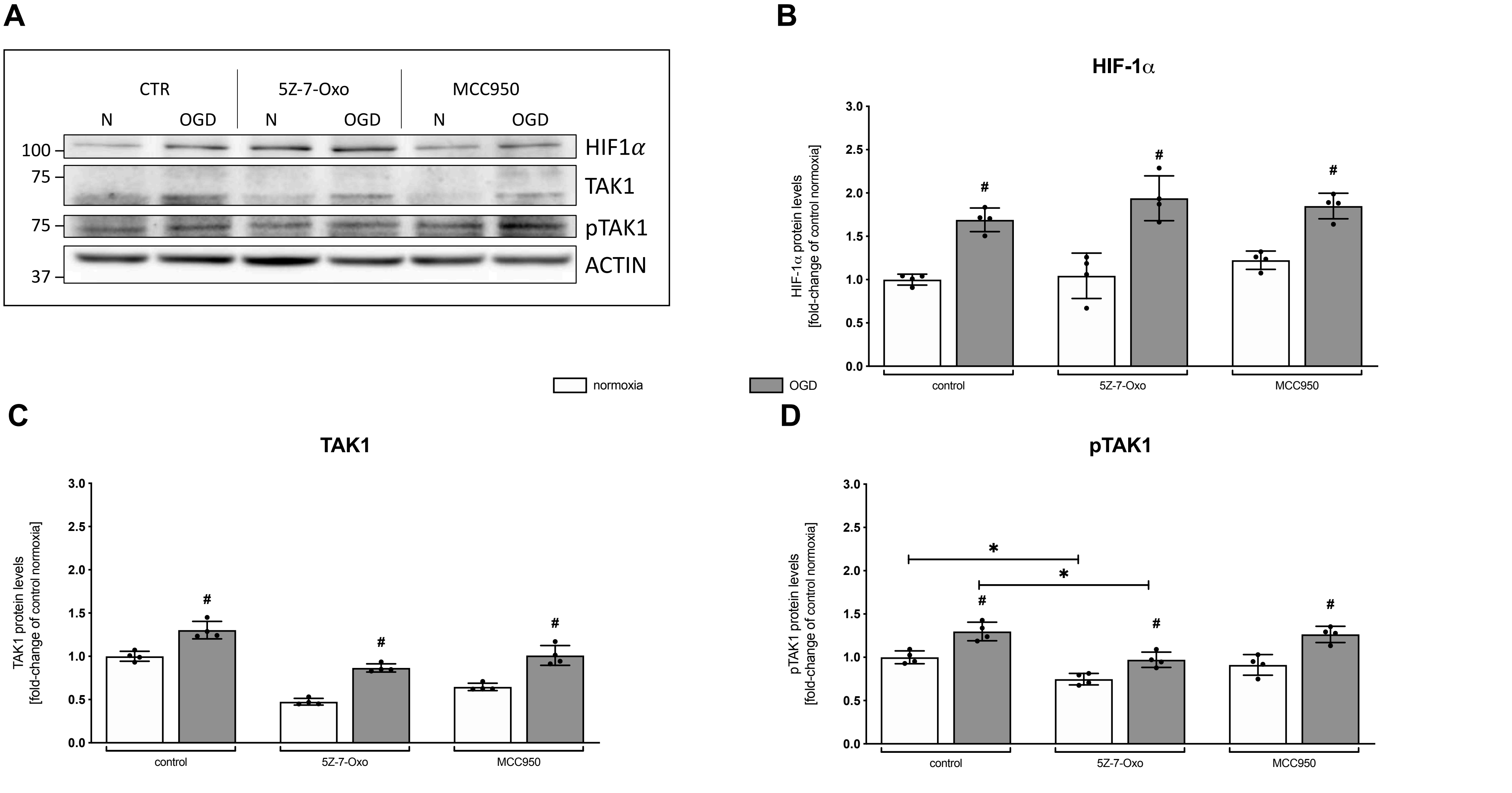

Supplement: Supplementary file 1 — Intraoperative laser doppler flowmetry revealed no differences between genotypes. (A) Schematic illustration of the treatment protocol for both genotypes, 4HT injections and EPO as well as NaCl treatment. (B) Scheme of protocol summarizing the number of total animals (64 mice). Exclusion criteria are described in material and methods section. (C) Changes in ipsilateral Laser Doppler flowmetry by intraluminal during MCAO procedure were monitored. The baseline blood flow was considered as 100% for all mice. Changes in LDF after occlusion of CCA, MCA and reperfusion are demonstrated. Abbreviations: bp: base pairs; CCAo: Common Carotid Artery occlusion; MCAo: Middle Cerebral Artery occlusion; LDF: Laser Doppler flowmetry. Online Resource 2. Immunohistochemical analysis of the NLRP3, NLRC4 and AIM2 inflammasomes in microglial cells, astrocytes and neurons after acute ischemic stroke. Immunofluorescence of brain slices after 30 min of tMCAo and 72 h of reperfusion in Tak1fl/fl and Cx3cr1creER –Tak1fl/fl treated with NaCl or EPO. (A + E) Representative images of double staining of a inflammasome such as NLRP3, NLRC4 or AIM2 (green) and a cell marker like NeuN, GFAP or Iba1 (red); nuclei were counterstained with DAPI (blue). (B-D + F-H) Double positives cells per 100 cells in the periinfarct zone are given in percentage. Data are presented as mean ± SD. Differences were tested by Mann-Whitney (n = 4). A list of used Antibodies is given in Table 3. Online Resource 3. In vitro stroke model, cell viability and IL-1β protein levels in murine microglial cells. (A) Schematic illustration of cell cultivation and treatment protocol of BV-2 cells for viability assessment. (B) Environmental conditions during hypoxia in the chamber and the supernatant. (C + D) LDH release in supernatant in % of lysis control during normoxia and after OGD with different concentrations of 5Z-7-Oxozeaenol and MCC950. (E) LDH release in the supernatant in % of lysis control during normoxia and after OGD a [file 12975_2021_948_MOESM1_ESM.pdf]
